# Automated scoring of the autobiographical interview with natural language processing

**DOI:** 10.3758/s13428-023-02145-x

**Published:** 2024-01-17

**Authors:** Ruben D.I. van Genugten, Daniel L. Schacter

**Affiliations:** 1 Institute for Experiential Artificial Intelligence, Northeastern University, Boston, MA USA; 2 Department of Psychology, Harvard University, Cambridge, MA USA

**Keywords:** Autobiographical interview, Autobiographical memory, Automated scoring, Natural language processing, Large language models

## Abstract

The autobiographical interview has been used in more than 200 studies to assess the content of autobiographical memories. In a typical experiment, participants recall memories, which are then scored manually for internal details (episodic details from the central event) and external details (largely non-episodic details). Scoring these narratives requires a significant amount of time. As a result, large studies with this procedure are often impractical, and even conducting small studies is time-consuming. To reduce scoring burden and enable larger studies, we developed an approach to automatically score responses with natural language processing. We fine-tuned an existing language model (distilBERT) to identify the amount of internal and external content in each sentence. These predictions were aggregated to obtain internal and external content estimates for each narrative. We evaluated our model by comparing manual scores with automated scores in five datasets. We found that our model performed well across datasets. In four datasets, we found a strong correlation between internal detail counts and the amount of predicted internal content. In these datasets, manual and automated external scores were also strongly correlated, and we found minimal misclassification of content. In a fifth dataset, our model performed well after additional preprocessing. To make automated scoring available to other researchers, we provide a Colab notebook that is intended to be used without additional coding.

**Supplementary Information:**

The online version contains supplementary material available at 10.3758/s13428-023-02145-x.

## Introduction

The autobiographical interview (interview) (Levine et al., 2002) is a widely used method to study the contents of participants’ autobiographical memories. In a typical experiment using the interview, participants are asked to recall a specific event for each cue, making sure to report as much detail as they can. Several human raters then use a manual to identify and count internal details (central episodic details) and external details (mostly non-episodic details) in the narratives. Levine et al. (2002) first developed the interview and its scoring manual to study age-related differences in memory. With this procedure, Levine et al. (2002) showed that older adults provide fewer internal details than young adults when they were asked to retrieve autobiographical memories that are more than a year old, despite being able to provide the same number of external details. For memories that were less than a year old, older adults provided more external details than younger adults. These findings extended our understanding of the effect of aging on autobiographical memory and in so doing, also highlight that the interview can be used to study group differences in memory.

Studies conducted since Levine et al. (2002) have made it clear that the interview enables researchers to test specific theories about memory. For example, Addis et al. (2008) hypothesized that if episodic retrieval supports the construction of specific future events, age-related decreases in internal details during retrieval of past events should be accompanied by age-related decreases in internal details when imagining future events. Addis et al. (2008) adapted the interview to ask participants about imagined future events and remembered events. In addition to finding support for their hypothesis, they found a positive correlation between the number of internal details participants provided when remembering the past and imagining the future. These results are consistent with the constructive episodic simulation hypothesis, which suggests that we imagine future experiences by recombining elements from episodic memories (Schacter & Addis, 2007). Other researchers have similarly adapted the interview to study future thinking. For example, Race et al. (2011) studied amnesic individuals with the interview, and showed that damage to the medial temporal lobe led to reductions in central episodic details on both episodic memory and future thinking tasks, despite intact descriptive abilities (i.e., normal performance when describing pictures).

Because the interview can be used to study features of autobiographical memory and future thinking, the procedure has seen widespread use across domains of psychology. In addition to the aforementioned studies of aging and amnesia, it has been used to study Alzheimer’s disease (e.g., Irish et al., 2011), depressive disorders (e.g., Söderlund et al., 2014), and the contributions of episodic retrieval to other domains of cognition, such as means–ends problem solving (e.g., Madore & Schacter, 2014). As of November 2021, over 200 studies have used this interview (Levine, 2021), and the paper that first described the interview has over 1400 citations listed on Google Scholar.

The autobiographical interview is widely used in psychology research even though scoring the interview takes considerable time and effort (typically 10 min per memory, and each participant typically provides multiple memories). Having to manually annotate hundreds of pages of memories potentially limits the interview's usefulness and breadth of applications. Because large studies using the interview are impractical, researchers have typically studied only effects that could be detected with small samples (e.g., approximately 30 participants).

Here we introduce an automated scoring procedure for the interview that can reduce experimenter burden and help researchers to conduct larger experiments and study smaller effects. We believe that this new procedure will broaden the scope of research questions the field can address. Our automated scoring approach could also make online data collection more practical. Online data collection is rarely used with the interview, likely because large numbers of participants are needed to compensate for noisy data. Online studies would allow researchers to gain access to a larger and more diverse population than the typical samples that have been used.

In the remainder of this paper, we will describe how narratives are typically scored, how researchers have attempted to streamline scoring, and our new approach for automating interview scoring.

### Current approach for manually scoring memory details

Researchers follow a set of rules from the interview manual to score narratives. These rules explain how to classify pieces of text as internal or external, and how to identify bits of information within these segments that count as details. Internal details refer to episodic details, and external details refer to non-episodic details (or episodic details that do not correspond to the central event being remembered or imagined). Internal details describe components of an event that are specific to time and place. The event’s location and time, the people, objects, actions, thoughts, and perceptual details involved are all internal details. External details, on the other hand, are largely non-episodic details. These are any details that do not belong in the internal details category, and mainly consist of factual information that does not require the participant to remember or imagine a specific event (e.g., “I’ve always enjoyed going to the beach for my birthday”). Participants sometimes provide information about events other than the central event they are being asked to describe. These details, while episodic in nature, are considered external details as well. Lastly, repetitive information (e.g., someone describes the same thing twice), and information unrelated to the event the participant is trying to describe (e.g., ‘sorry for that cough!’) are also considered external.

The manual for the interview provides clear rules on how to divide up segments of text into individual details. For internal segments, each piece of information that tells us something more about the event is generally counted as a detail. For example, “he had a hat” is considered one detail. Any additional descriptors count as additional details, e.g., “he had a brown hat” is considered two details. This description is illustrative of the general approach; for an exhaustive list of rules and exceptions, see the manual available upon request from Dr. Brian Levine (blevine@research.baycrest.org). A brief scoring example is provided in the appendix.

### Existing automation approaches for memory scoring

Several researchers have streamlined scoring of the interview, yet no group has fully automated the scoring of details in narratives. Previous work consists of two approaches: speeding up the processes involved in scoring, and predicting the number of internal and external details. For example, Wardell et al. (2021a) automated the process of transcribing spoken narratives to text with Dragon NaturallySpeaking software. The researchers also reduced the time necessary for scoring by setting up keyboard shortcuts in Microsoft Word. Once details were manually scored, their software automatically counted the scored details in each memory. After implementing a protocol of this kind, a research group would be able to score more rapidly (see, e.g., Wardell et al., 2021b). However, much of the work remains to be done by hand: identifying internal and external content and separating the narratives into details are both still done manually.

To the best of our knowledge, only one paper reports an attempt to automatically generate autobiographical interview detail scores for each narrative. Peters et al. (2017) first extracted 83 features from each narrative, such as the number of emotion words, the valence of these emotion words, the number of words in the story, and the number of nouns in the story. Peters et al. then used these features in several regression models (e.g., principal component regression) to predict the number of internal and semantic details each participant provided (summed across five or 12 narratives per participant). When Peters et al. predicted the number of internal details provided by each participant, root-mean-square error (RMSE) was approximately .5 for internal details and .65 for semantic details. Peters et al. report one model built to predict the number of internal details in individual narratives. RMSE for this model was approximately .75 for episodic future thinking narratives and .85 for autobiographical memory narratives. No model was reported that predicted semantic details in individual narratives. To contextualize these results, a simple model that predicts the mean number of internal or semantic details for every narrative in this dataset would result in RMSE = 1, while a model with perfect predictions would result in RMSE = 0. Importantly, word count was a significant predictor for models predicting internal and semantic details counts. Since predictions were driven in part by the total amount of content, these regression models are presumably misclassifying internal content as semantic, and vice versa. So, while these researchers took an important first step by attempting to automatically score the interview, their predicted memory scores differed significantly from the actual memory scores, and additional work is needed to automate interview scoring.

Related work (Takano et al., 2017, 2018, 2019) has automated the scoring of the simpler autobiographical memory test (AMT) (Williams & Broadbent, 1986) with more success. When using the autobiographical memory test, human raters classify memories as specific or general. Takano et al. used word frequencies and parts of speech frequencies to train a classifier to determine which memories were specific and which memories were general. Across studies, Takano et al. report good classification results, with high accuracy, frequent correct identification of specific memories, and frequent correct identification of general memories. For example, for narratives from English-speaking adults reported in Takano et al. (2019), classification was 81.1% accurate. Specific memories were correctly identified in 81.8% of cases, and general memories were correctly identified in 80.3% of cases. These results suggest that natural language processing provides a promising path for automated memory analyses. In this paper, we tested whether natural language processing can be used to automate the more complex scoring procedure of the autobiographical interview.

### Our automated scoring approach

To improve automated scoring accuracy of the interview, we relied on advances in natural language processing to identify the amount of internal and external content in each sentence of an interview narrative. After classifying each sentence, we counted the amount of internal and external content in each narrative and validated these counts against detail counts obtained through manual scoring.

We trained our classifier by using data scored according to the interview. Specifically, we used these data to fine-tune weights of an existing neural network, which had previously been trained on different natural language tasks. This procedure allowed us to take advantage of the language representations that the neural network had previously learned. This process, known as *transfer learning*, is a standard approach for classifying language content according to new labels, especially when few training examples are available (for introduction, see e.g., Azunre, 2021). Specifically, we fine-tuned distilBERT (Sanh et al., 2019) with the ‘huggingface’ library (Wolf et al., 2020).

We trained and evaluated our model with five datasets, which involved data scored according to the standard or adapted interview. We found that our code accurately identified internal and external content, with minimal misclassification of internal content as external, and minimal misclassification of external content as internal.

## Methods

### Model training and evaluation data

To train our model to classify the amount of internal and external content in sentences, we requested data from several different researchers. All data we used were previously scored on a computer using standard or adapted interview scoring manuals. These data spanned several different tasks. Three datasets contained autobiographical memories (King et al., 2022; Sheldon et al., 2020; Strikwerda-Brown et al., 2021), and one of these contained data from both younger and older adults (Sheldon et al., 2020). Another dataset contained future simulation data from younger and older adults (Devitt & Schacter, 2018, Devitt & Schacter, 2020). We also included data from a study on creative writing (van Genugten et al., 2021) that was scored using an adapted interview scoring manual. These data were included to test whether the model would generalize to a non-memory or future simulation paradigm that used adapted interview scoring. Last, one dataset included a picture description task and an open-ended thoughts description task (Strikwerda-Brown et al., 2021). These data were scored with guidelines that were different from the adapted or standard interview manuals. So, these data were included for exploratory analyses, without the expectation that our model would perform well on them. Because they were scored differently from all the other datasets, they were never included in the training sets. Each of these datasets is described in more detail below.

#### Dataset 1: Autobiographical memories (King et al., 2022)

King et al. (2022) examined how retrieving memories from an observer perspective (as opposed to a first-person perspective) changed the narratives. In the first session of this study, participants were asked to elaborate on a subset of memories in which they rated the event as occurring through their own eyes (at least a 5 on a seven-point scale measuring self-perspective). We used these memories for our analyses. These data were in written form and were scored according to the interview (Levine et al., 2002). Scoring reliability was high; Cronbach’s alpha was greater than .88 for both internal and external detail counts.

This study generated a dataset of 40 individuals (25 female). Participants were, on average, 23.33 years old (SD = 3.17). All participants indicated that they were not previously diagnosed with a mood or cognitive disorder, nor taking any medication that could affect performance on the study. All participants were recruited from the Harvard study pool and the community.

#### Dataset 2: Autobiographical memories (Sheldon et al., 2020)

Sheldon et al. (2020) collected autobiographical memories to test whether cue valence and arousal affected subsequent retrieval and elaboration of memories. In this experiment, participants listened to a series of 24 musical excerpts, which served as retrieval cues. After each retrieval cue, participants wrote down a caption to describe the memory they had retrieved. In a second session, participants were presented with the captions they had previously written down, given 30 s to recall the memory, and then used two minutes to describe what they remembered. Responses were audio-recorded and transcribed. Responses were then scored using the standard scoring guidelines from the interview (Levine et al., 2002). Three raters were assessed for reliability; correlations between internal details and correlations between external details were greater than .8.

Participants were recruited from McGill University’s study pool. Each of the 42 participants was fluent in English and free of major neurological or psychiatric disorders. Participants were on average 20 years old (SD = 1.4) and had 14.6 years of education (SD = 1.1). Thirty-seven of the participants were female.

#### Dataset 3: Autobiographical memories, thoughts, and picture descriptions (Strikwerda-Brown et al., 2021)

Strikwerda-Brown et al. (2021) investigated age-related changes in memory on a cued retrieval task and an open-ended task. Participants also completed a picture description task (cf., Gaesser et al., 2011). On each trial of the experiment, the participants saw a picture, were asked to retrieve a memory related to the image (memory task), to describe what was present in the image as if to someone who could not see the image (description task), or to describe the thoughts that arise when viewing the picture (thoughts task). All narratives were transcribed after being verbally reported by the participants.

Scoring of the memory task followed guidelines developed in Levine et al. (2002), with modified scoring guidelines for external details as described in Strikwerda-Brown et al. (2019). The picture description task was scored by following guidelines developed by Gaesser et al. (2011). Perceptual details in the picture were scored as internal details, and all other details (e.g., inferences about the picture, general comments about the picture) were scored as external. Details in the thoughts task were considered internal if they described any past event; all other details were considered external. To assess inter-rater reliability, intraclass correlations were computed and were greater than .85 for internal and external detail categories.

Twenty-four older adults and 25 younger adults were included in the analysis of this study. Younger adults were, on average, 21.7 years old (SD = 2.4). Participants reported no neurological or psychiatric impairments that would affect the study. Older adults were recruited from an existing database of older adults in the Montreal area. Younger adults were recruited from the McGill University study pool and surrounding areas.

#### Dataset 4: Future simulation: Young adult and older adult data (Devitt & Schacter, 2018; Devitt & Schacter, 2020)

Devitt & Schacter (Devitt & Schacter, 2018, Devitt & Schacter, 2020) examined how episodic simulation of an event before learning of its outcome affects the subsequent memory of that outcome. In their studies, participants were presented with a series of cues for future events and were instructed to imagine the events going well or poorly for 3 min. Participants were instructed that each imagined event should occur within the next year. Afterwards, participants were given descriptions of how the events happened. In a second session, participants were tested for their memory of how the event happened.

Across two studies, future simulations from older and younger adults were audio-recorded, transcribed, and scored according to the interview. To calculate reliability, standardized Cronbach’s alpha was computed using a mixed model; Cronbach’s alpha was .91 for internal details and .79 for external details. Data from these experiments include 27 younger adults (mean age = 22.59 years, SD = 3.18, 12 male) and 25 older adults (mean age = 72.24, SD = 6.49; seven male). Participants indicated no history of neurological or psychiatric impairment. These participants were recruited from Harvard University and the surrounding community, using the Harvard psychology study pool.

#### Dataset 5: Creative writing narratives (van Genugten et al., 2021)

van Genugten et al. tested whether episodic retrieval contributes to creative writing performance. Specifically, van Genugten et al. used the Episodic Specificity Induction (Madore et al., 2014; for review, see Schacter & Madore, 2016) to manipulate episodic retrieval prior to a creative writing task. Detail counts after the ESI were compared to detail counts after two control inductions.

In the creative writing task, participants read a series of excerpts from literature and were asked to continue writing each story in a style that felt natural to them. Each story was scored according to scoring guidelines from the ESI studies of Madore et al. (2014) and Jing et al. (2016), which were adapted from the standard interview scoring (Levine et al., 2002). In their scoring, van Genugten et al. also considered all event details as internal details. This procedure differs from previous guidelines, which only considered details from the central events to be internal. This change was made to ensure that no episodic details were marked as external. Scorers achieved high reliability (Cronbach’s alpha = .93 for internal details and .90 for external details). Data from the first experiment were scored by hand, and as such were not used in the training and evaluation of our model. Data from the second experiment of this study were used since scoring was done on the computer.

Data used in this paper came from 32 participants, who each wrote ten stories. Participants were young (18–30 years old, M = 24.03 years, SD = 3.51; 21 female, 11 male) and recruited from the Harvard University study pool. No participant reported neurological or psychiatric impairment at the time of the study.

### Data preparation

Data were read in from various sources, including text files and SciTos (Wickner et al., 2015) html exports. Any prompting by the researcher (e.g., ‘tell me more about that’) was removed. Data were manipulated so that formatting was identical across datasets. Because our approach classifies individual sentences, narratives were split into sentences using pySBD (Sadvilkar & Neumann, 2020). pySBD splits text into sentences based on 48 rules that rely in part on punctuation.

Additional preprocessing was necessary after we noticed that some exceptionally long sentences contained a large majority of the narrative they came from (or even the full narrative). These narratives were transcribed with little or no punctuation, leading to few sentence splits by pySBD. To mimic narratives transcribed with full punctuation, we removed sentences that contained more than eight details, since these sentences are likely missing punctuation. Detail counts associated with the narratives, which we used for validation, were updated to reflect the removal of this content. Additional details on this preprocessing are discussed in the supplemental materials. This preprocessing step is not included in the code we make available that other researchers can use to automatically score their own narratives. Researchers who want to use our model in their own research should add punctuation as they are transcribing, to accommodate sentence splitting by pySBD. Alternatively, participants can be asked to type narratives, so that researchers do not have to add punctuation as they transcribe.

Each sentence was classified as belonging to one of four categories: containing 0% internal content (i.e., 100% external content), 50% internal content, 75% internal content, or 100% internal content. We modified training datasets such that there were an equal number of sentences in all four categories. We did this by identifying the category with the greatest number of sentences, and upsampling data from all other categories. So, for example, if a training dataset were to contain 10,000 fully internal sentences, and 8000 sentences from each of the three remaining categories, we would sample 2000 sentences with replacement from each of those three categories, then add those sentences to the dataset so that we have 10,000 training examples in each category. We used training data with an equal number of examples for each label because this procedure prevented the model from learning to use relative frequencies of internal and external details to improve prediction accuracy. This step is necessary because if we did not upsample our training sets, and our narratives contained many more internal details than external details, the model could obtain relatively high accuracy by classifying all details as internal.

### Model training and evaluation

We trained and evaluated the performance of our classification model with five datasets that are described in more detail in *Model Training and Evaluation Data*. We iteratively left out one dataset for evaluation, using the other four for training. For some datasets, data from multiple tasks or experiments were available. When these datasets served as the testing set, performance on each task was separately evaluated. For example, one dataset involved future simulation for older adults and younger adults. When this dataset was left out for evaluation, the model was trained on the other four datasets and was then separately evaluated on the older adult data and the younger adult data. We report performance of all evaluation sets separately. Picture description and thoughts tasks from Strikwerda-Brown et al. (2021) were never included in training data because they were not scored with the adapted or standard interview.

We trained our model to classify the proportion of each sentence that consisted of internal and external content. To generate automated scores for each new narrative, we used this model to predict the amount of internal and external content in each sentence in the narrative, then summed these predictions to obtain total scores for the narrative. For ease of modeling, we used near-perfect proxies for the number of internal and external details in our approach: the number of words in internal segments, and the number of words in external segments. That is, each narrative was summarized by the number of internal words and the number of external words in the narrative. Below, we outline our approach in more detail.

#### Classifying information as internal or external: Overview

To identify internal and external information, we adapted a common approach for classifying text. We fine-tuned weights of an existing neural network with new data. We trained our model to classify sentences as containing only external content, 50% internal content, 75% internal content, or 100% internal content. To select classification labels, we calculated the percent of internal content in each sentence in the first dataset we obtained (Devitt & Schacter, 2018; Devitt & Schacter, 2020). A histogram of these percentages showed clusters at approximately 0, 50, 75, and 100%; hence, our labels.

#### Classifying information as internal/external: Model specifics

To identify internal and external information, we used a model designed to be fine-tuned on text for classification. Specifically, we used distilBERT (Sanh et al., 2019), which is a large language model that can be fine-tuned to new tasks by adding a classification head. The classification head contains a single linear layer for classification at the end of the network’s pooled output. Fine-tuning this model involves changing the weights of the network to improve predictions on the fine-tuning data. distilBERT provides strong performance on a range of natural language processing benchmark tests, while using fewer parameters than its ancestor BERT (Devlin et al., 2019). distilBERT has been trained to mimic BERT’s performance on two tasks: masked word prediction and next sentence prediction. Training with next sentence prediction involves providing the model with pairs of sentences. For each pair of sentences, the model must determine whether the second sentence followed the first sentence in the source text, or whether that pair of sentences is randomly paired. Training with masked word prediction involves randomly masking a subset of words in each sentence (e.g., ‘the [MASK] gave the soccer player a yellow card’), then training the model to predict what the masked words are (‘referee’ in this case). Both types of learning require no human annotation but allow the network to acquire language knowledge that can then be taken advantage of in subsequent fine-tuning. Training data for these prediction tasks come from English Wikipedia text and the BookCorpus (a dataset of 11,038 unpublished books). We chose to use distilBERT instead of BERT or RoBERTa (Liu et al., 2019) because of its rapid training, as our model had to be trained six times: five times for our leave-one-dataset-out cross validation, and once for training on all datasets together.

We used Huggingface Transformers (Wolf et al., 2020) to fine-tune distilBERT on our classification task. We used accuracy as our evaluation criterion when training. We used Huggingface’s default training arguments for fine-tuning. ﻿We used three training epochs, a batch size of 16 per device during training, a batch size of 64 for evaluation, 500 warmup steps, and a .01 weight decay.

#### Separating text into details

The interview scoring manual provides guidelines on how to split text into individual details, which allows researchers to quantify how much internal and external content is present in narratives. For ease of modeling, we take a different approach to quantifying internal and external content. Instead of splitting sentences into individual details, we aim to instead predict the number of internal words and external words in each sentence. In our analyses, which we discuss below, we found that the number of words in internal segments is a near-perfect proxy for the number of internal details. Likewise, we found that the number of words in external segments is a near-perfect proxy for the number of external details.

To establish whether internal word count adequately captures the number of internal details in narratives, we examined the correlation between these two variables across our datasets. The correlation between the number of internal details and internal word count ranged from .86 to .98 (mean = .92). We repeated this process for external details. The correlation between the number of external details and external word count ranged from .87 to .98 (mean = .94). Together, these extremely high correlations suggest that we can use internal and external word counts to quantify the amount of internal and external information. That is, we do not need to split sentences into individual details for the purposes of this project. An example of scoring with internal and external details and internal and external word counts is provided in the Appendix. We also provide a more extended discussion on the relationship between detail counts and internal and external word counts there.

We should note that internal and external word counts may not be adequate for all circumstances. For example, splitting content into individual details may be helpful for researchers that want to assign each detail to a subcategory (e.g., place, time, perceptual, etc.). However, because the purpose of this project is simply to quantify the amount of internal and external content in narratives, we used internal and external word count as excellent approximations of internal and external details.

### Evaluating model performance

**Fig. 1:**
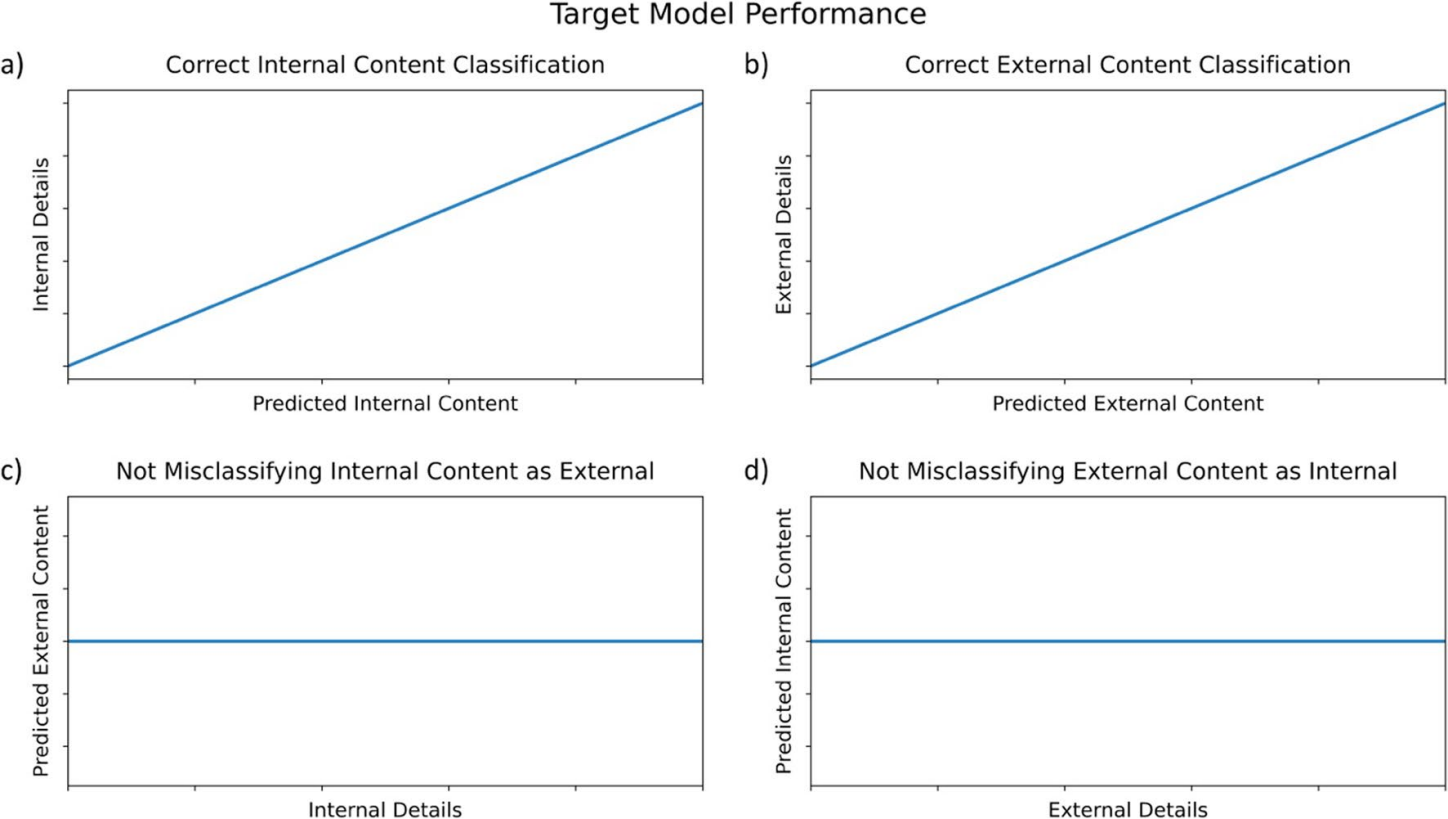
Target model performance. Internal (**a**) and external (**b**) content are accurately identified, with no misclassification of internal details as external (**c**) and external details as internal (**d**)

After classifying each of our sentences in the evaluation sets, we aggregated all sentences for each narrative and obtained an estimate of the amount of internal and external content in each narrative. We correlated these estimates with internal and external detail counts for validation. If our model were successful, we would expect the predictions of internal and external content to match actual internal and external detail counts. Our expectations, then, were that (1) the number of internal details in the narratives would correlate with the amount of predicted internal content; (2) the number of external details in the narratives would correlate with the amount of predicted external content. Because the purpose of the interview is to correctly label content as internal or external, we also expected the model to not misclassify internal content as external. In other words, we also expected that (3) the number of internal details would be unrelated to the amount of predicted external content. Likewise, we should not misclassify external content as internal, so we further expected that (4) the number of external details would be unrelated to the amount of predicted internal content. These four predictions are displayed graphically below in Fig. 1. For each evaluation dataset, we report results in a similar format.

## Results

To evaluate how well our model scored narratives, we examined internal and external detail scores as a function of predicted internal and external content. We evaluated performance on each left-out dataset separately.

### Narratives scored with the standard or adapted interview

#### Results: Future simulation: Older adult data (Devitt & Schacter, 2018; Devitt & Schacter, 2020)

**Fig. 2:**
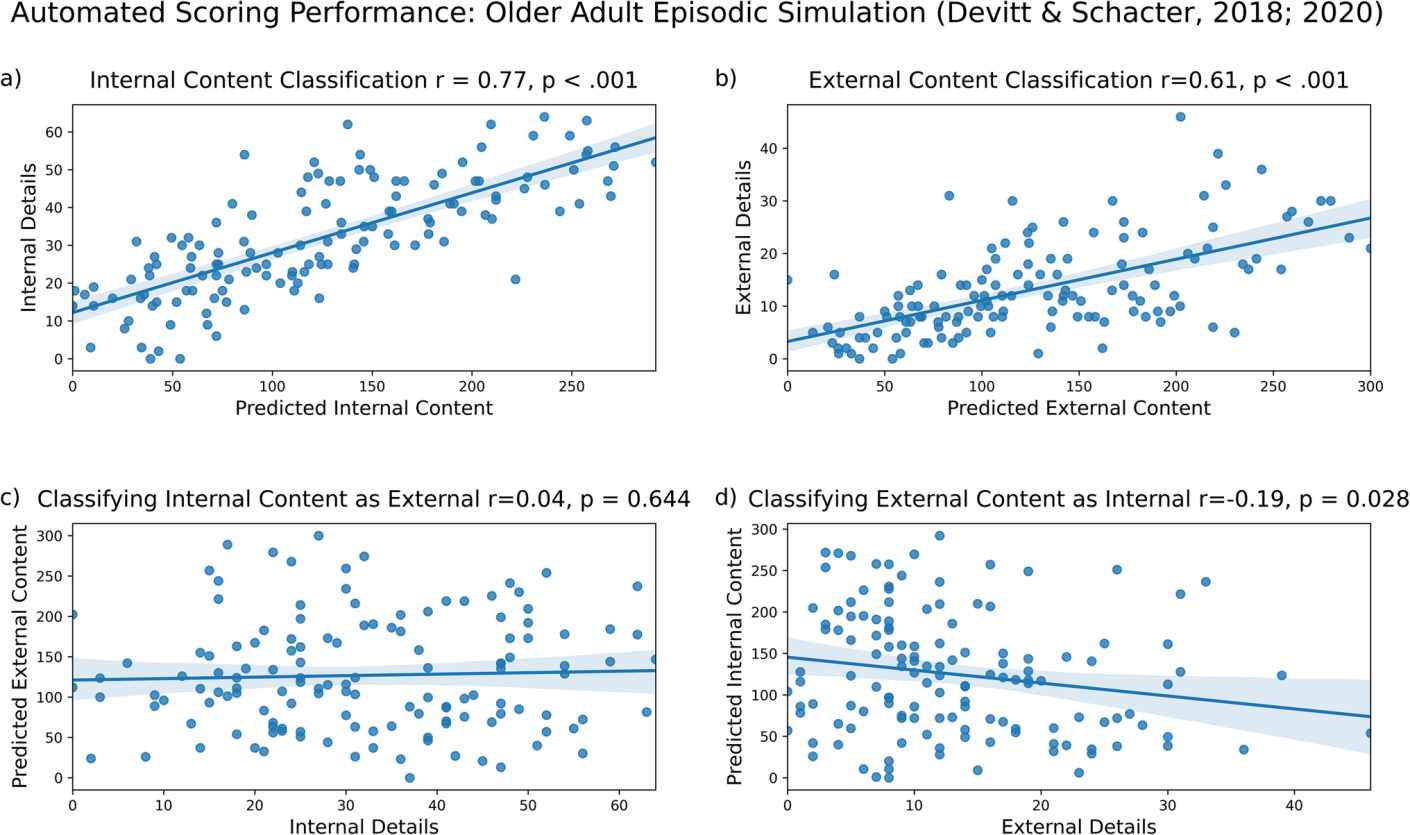
Model performance on older adult episodic simulation data from Devitt and Schacter (2018, 2020). Internal (**a**) and external (**b**) content are accurately identified, with minimal misclassification of internal details as external (**c**) and external details as internal (**d**)

We examined whether our model correctly identified internal content. We found a strong relationship between predicted internal content and the number of internal details in future simulation narratives (Fig. 2a, *r* = .77, *p* < 0.001). We also examined the extent to which our model correctly identified external content. We found a strong relationship between predicted external content and the number of external details in future simulation narratives (Fig. 2b, *r* = .61, *p* < 0.001). We expected to find lower correlations when we examined the extent to which our model misclassified data. We examined how much internal content was misclassified as external content by our model. We did not find a significant relationship between internal details and predicted external content (Fig. 2c, *r* = .04, *p* = 0.644). We also examined how much external content was misclassified as internal content. We found a weak negative relationship between external details and predicted internal content (Fig. 2d, *r* = – .19, *p* = .028). To summarize, we found greater correct classification of internal content than misclassification (*R*^2^ = 0.57 vs. *R*^2^ = 0.00). We also found greater correct classification of external content than misclassification (*R*^2^ = 0.37 vs. *R*^2^ = 0.04). These results are summarized in Fig. 2 below.

#### Results: Future simulation: Young adult data (Devitt & Schacter, 2018, Devitt & Schacter, 2020)

**Fig. 3:**
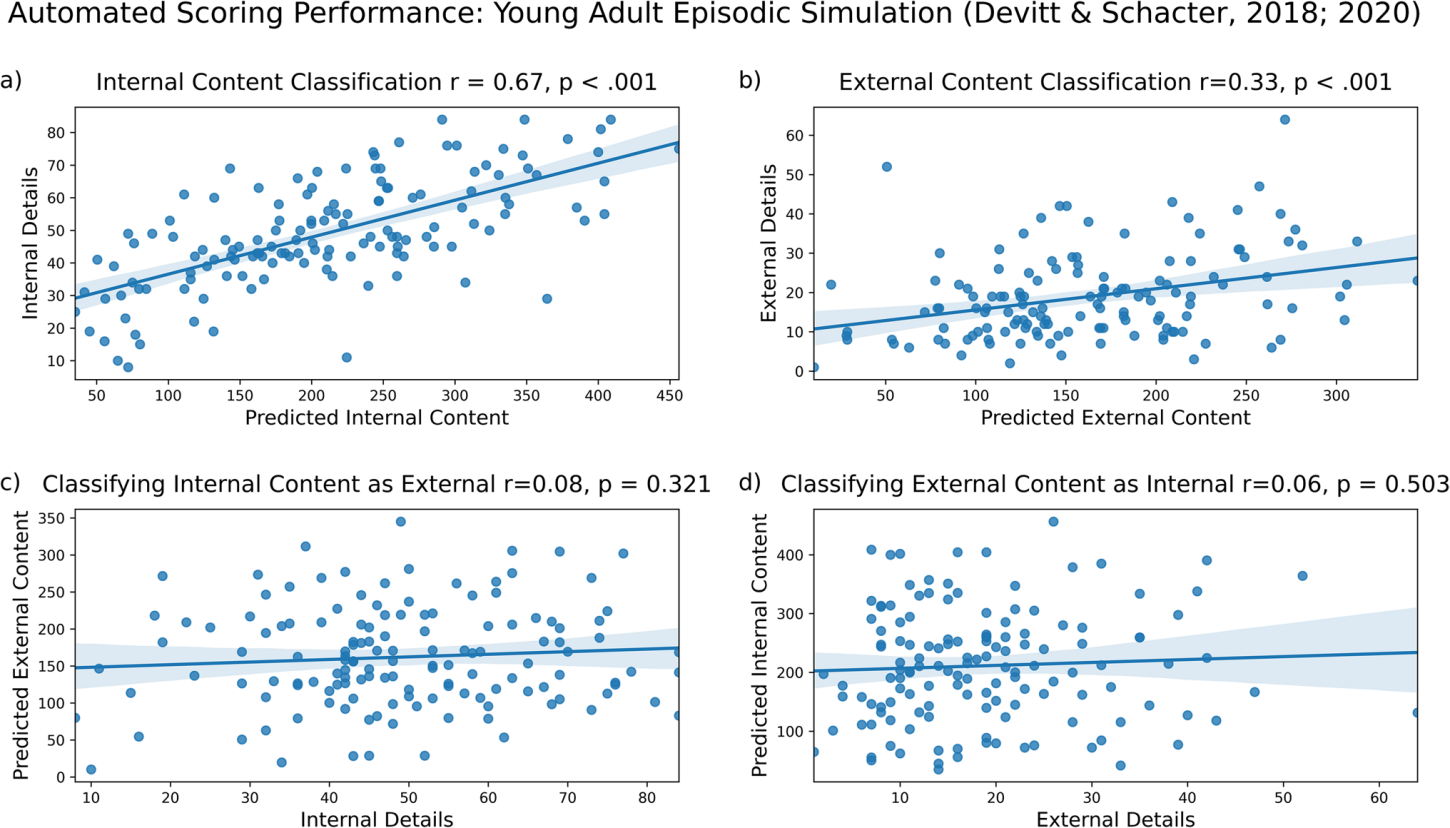
Model performance on young adult episodic simulation data from Devitt and Schacter (2018, 2020). Internal (**a**) and external (**b**) content are accurately identified, with minimal misclassification of internal details as external (**c**) and external details as internal (**d**)

Our model correctly identified much of the internal content (Fig. 3a; *r* = .67, *p* < 0.001) and also correctly identified some of the external content (Fig. 3b, *r* = .33, *p* < 0.001). As expected, we observed less misclassification than correct classification. Internal content was not significantly misclassified as external content (Fig. 3c, *r* = .08, *p* = 0.32), and external content was not significantly misclassified as internal content (Fig. 3d, *r* = .06, *p* = 0.503). To summarize, we found greater correct classification of internal content than misclassification (*R*^2^ = 0.44 vs. *R*^2^ = 0.01). We also found greater correct classification of external content than misclassification (*R*^2^ = 0.11 vs. *R*^2^ = 0.00). These results are summarized in the figure below.

#### Results: Autobiographical memory (King et al., 2022)

**Fig. 4:**
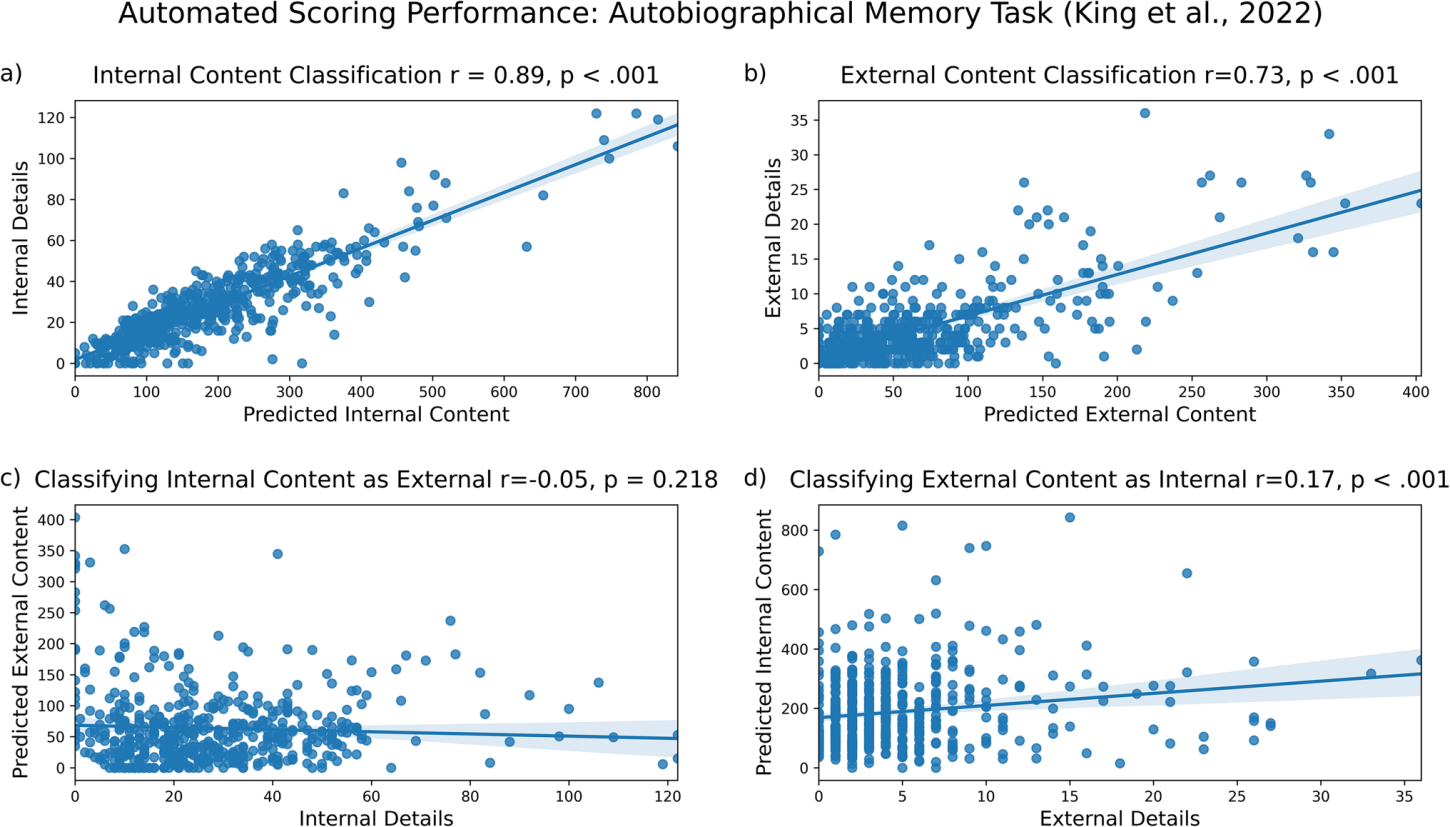
Model performance on autobiographical memory data from King et al. (2022). Internal (**a**) and external (**b**) content are accurately identified, with minimal misclassification of internal details as external (**c**) and external details as internal (**d**)

Once again, our model correctly identified much of the internal content (Fig. 4a; *r* = .89, *p* < 0.001) and also correctly identified much of the external content (Fig. 4b, *r* = .73, *p* < 0.001). As expected, we observed less misclassification than correct classification. Internal content was not significantly misclassified as external content (Fig. 4c, *r* = – .05, *p* = 0.218), and external content was not often misclassified as internal content (Fig. 4d, *r* = .17, *p* < 0.001). To summarize, we found greater correct classification of internal content than misclassification (*R*^2^ = 0.79 vs. *R*^2^ = 0.00). We also found greater correct classification of external content than misclassification (*R*^2^ = 0.53 vs. *R*^2^ = 0.03). These results are summarized in the figure below. In the Supplementary Materials, we present additional analyses which test for the effect of removing outliers on results. Those results are consistent with the findings presented here.

#### Results: Autobiographical memories (Strikwerda-Brown et al., 2021)

**Fig. 5:**
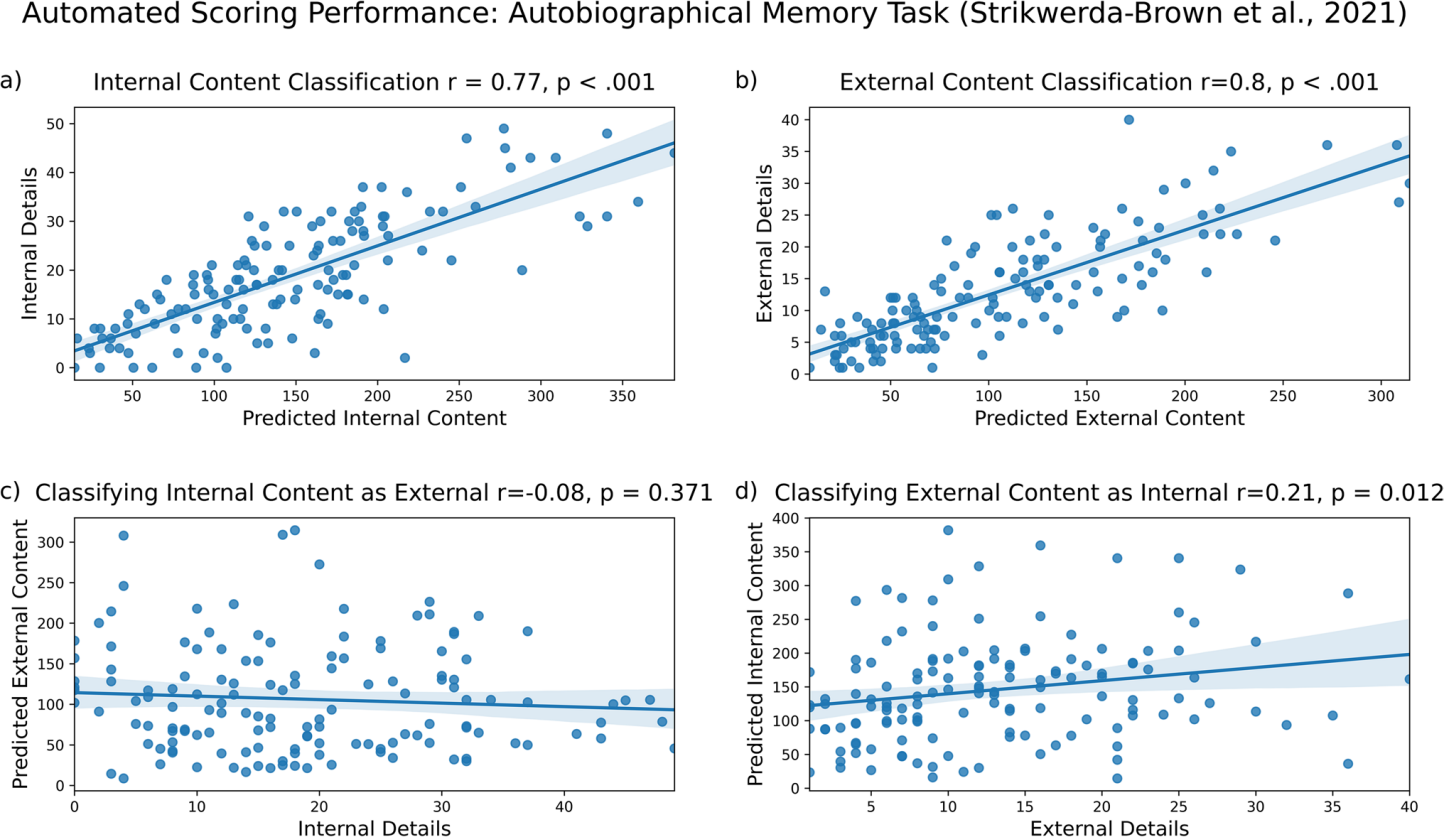
Model performance on autobiographical memory data from Strikwerda-Brown et al. (2021). Internal (**a**) and external (**b**) content are accurately identified, with minimal misclassification of internal details as external (**c**) and external details as internal (**d**)

Our model correctly identified much of the internal content (Fig. 5a; *r* = .77, *p* < 0.001) and external content in autobiographical memories (Fig. 5b, *r* = .80, *p* < 0.001). Internal content was not significantly misclassified as external content (Fig. 5c, *r* = – .08, *p* = 0.371), and external content was not often misclassified as internal content (Fig. 5d, *r* = .21, *p* = 0.012). To summarize, we found greater correct classification of internal content than misclassification (*R*^2^ = 0.59 vs. *R*^2^ = 0.01). We also found greater correct classification of external content than misclassification (*R*^2^ = 0.64 vs. *R*^2^ = 0.04). These results are summarized in the figure below.

#### Results: Creative writing narratives (van Genugten et al., 2021)

**Fig. 6:**
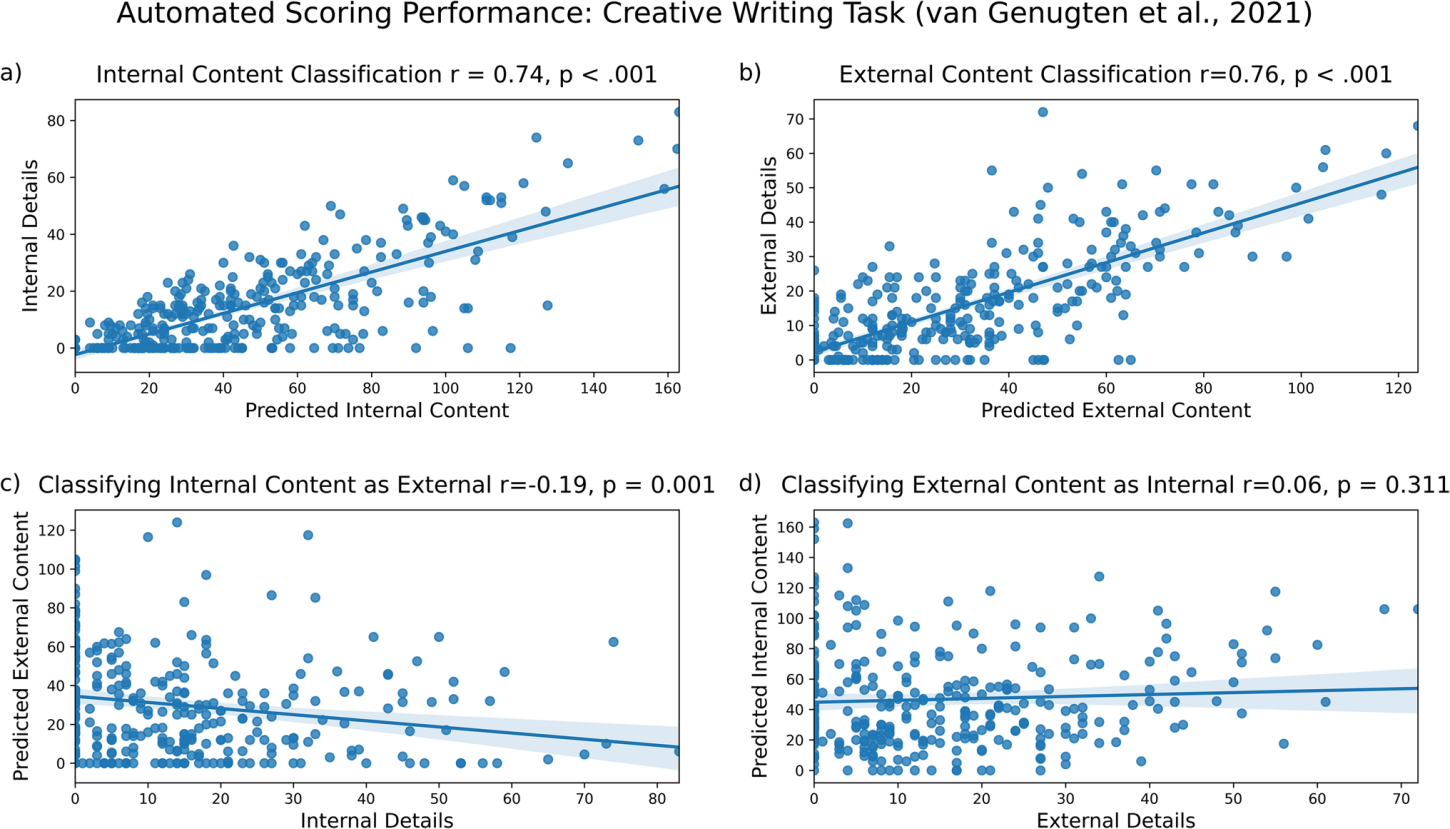
Model performance on narratives from van Genugten et al. (2021). Internal (**a**) and external (**b**) content are accurately identified, with minimal misclassification of internal details as external (**c**) and external details as internal (**d**)

Our model correctly identified much of the internal content (Fig. 6a; *r* = .74, *p* < 0.001) and external content (Fig. 6b, *r* = .76, *p* < 0.001) in creative writing narratives. As expected, we observed less misclassification than correct classification. Internal content was rarely misclassified as external content (Fig. 6c, *r* = – .19, *p* = 0.001), and external content was not significantly misclassified as internal content (Fig. 6d, *r* = – .06, *p* = 0.311). To summarize, we found greater correct classification of internal content than misclassification (*R*^*2*^ = 0.55 vs. *R*^*2*^ = 0.04). We also found greater correct classification of external content than misclassification (*R*^*2*^ = 0.57.vs. *R*^*2*^ = 0.00). These results are summarized in the figure below.

#### Results: Autobiographical memories (Sheldon et al., 2020)

**Fig. 7:**
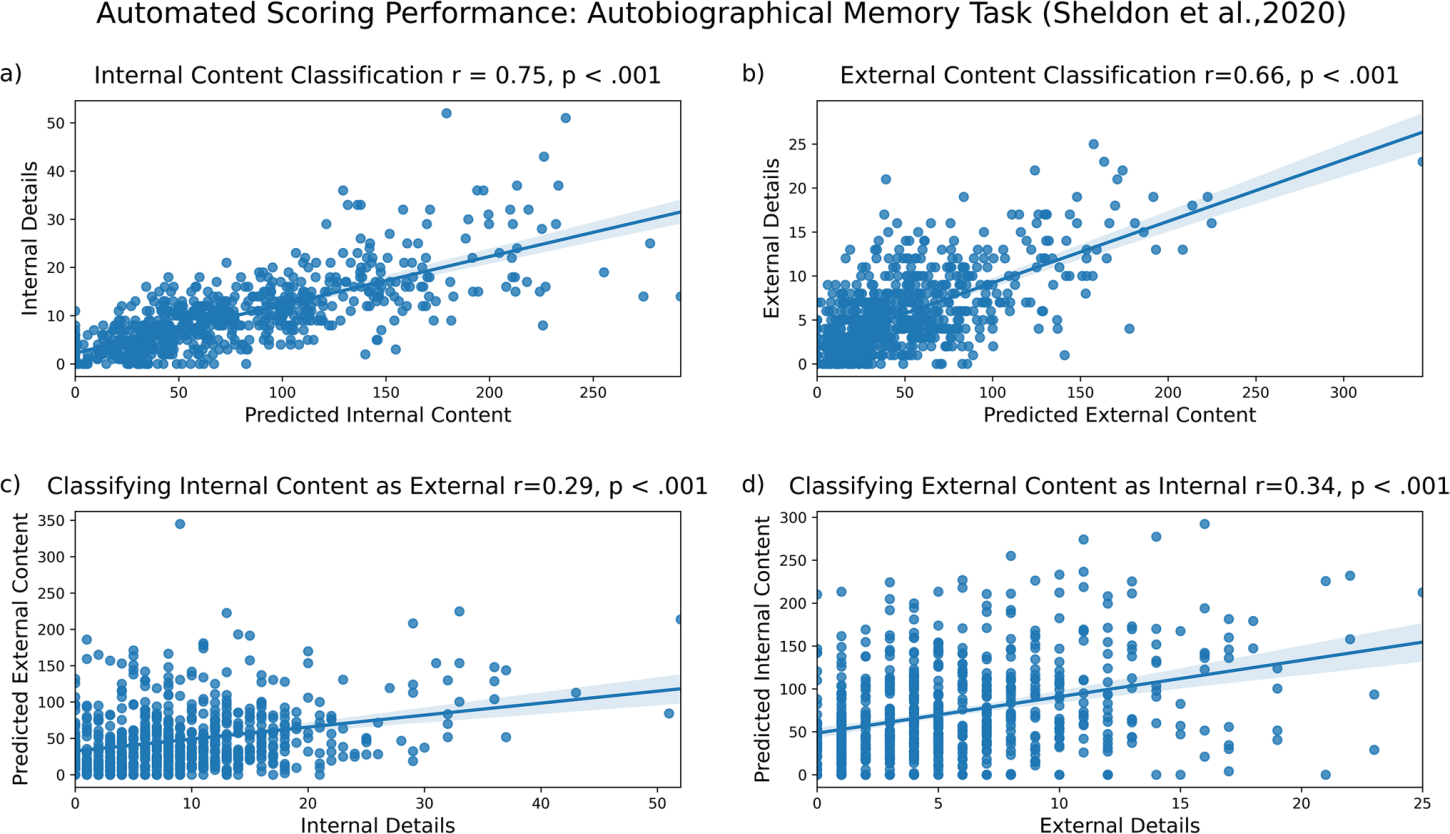
Model performance on autobiographical memory data from Sheldon et al. (2020). Internal (**a**) and external (**b**) content are accurately identified, with significant misclassification of internal details as external (**c**) and external details as internal (**d**)

Consistent with the previous analyses, our model correctly identified much of the internal content (Fig. 7a; *r* = .75, *p* < 0.001) and also correctly identified much of the external content (Fig. 7b, *r* = .66, *p* < 0.001). In contrast to previous results, we observed significant misclassification. Internal content was often misclassified as external content (Fig. 7c, *r* = .29, *p* < 0.001) and external content was often misclassified as internal content (Fig. 7d, *r* = .34, *p* < 0.001). Even though we found greater correct classification of internal content than misclassification (*R*^2^ = 0.57 vs. *R*^2^ = 0.08), misclassification rates were high. We also found greater correct classification of external content than misclassification (*R*^2^ = 0.44.vs. *R*^2^ = 0.12), but misclassification of external content is frequent. These results are summarized in the figure below. In the supplementary materials, we present additional analyses which test for the effect of removing outliers on results. Those results are consistent with the findings presented here.

We believe that several factors may be driving significant misclassification of internal details as external and vice versa. Many narratives in this dataset were transcribed (1) without much punctuation and (2) without removing uninformative speech (e.g., ‘I don’t know, we went to, I guess, well,’). These two factors do not affect manual scoring, but could significantly impact automated scoring. Narratives with little punctuation are problematic for our approach because it leads pySBD to split narratives into sentences incorrectly. Resulting segments of text contained multiple sentences, which makes accurate prediction difficult for the model. While our preprocessing removed very long pieces of text without punctuation, some pieces of text still contained multiple sentences. Likewise, uninformative speech is not a problem for manual scoring, but it could be problematic for our model because this text will be classified as internal or external content.

To explore whether our model would work better on this dataset if it had been transcribed with more punctuation, we manually added punctuation to a random subset of 100 narratives. We did not remove any sentences from these newly punctuated narratives before classification. We found that adding punctuation to the transcriptions led to correct identification of internal content with no detectable misclassification of internal content as external (*R*^2^ = .55 vs. *R*^2^ = .00; *r* = .74, *p* < 0.001 vs. *r* = .03, *p* = 0.734). Our model also correctly identified external content with some misclassification of external content as internal (*R*^2^ = .49 vs. *R*^2^ = .07; *r* = .70, *p* < 0.001 vs. *r* = .27, *p* = 0.006). We would expect even less content misclassification for this dataset if meaningless text were also removed. While these results are not perfect, they represent a significant improvement upon the non-punctuated analyses.

### Narratives scored with alternative scoring procedures

#### Results: Picture description task (Strikwerda-Brown et al., 2021)

**Fig. 8:**
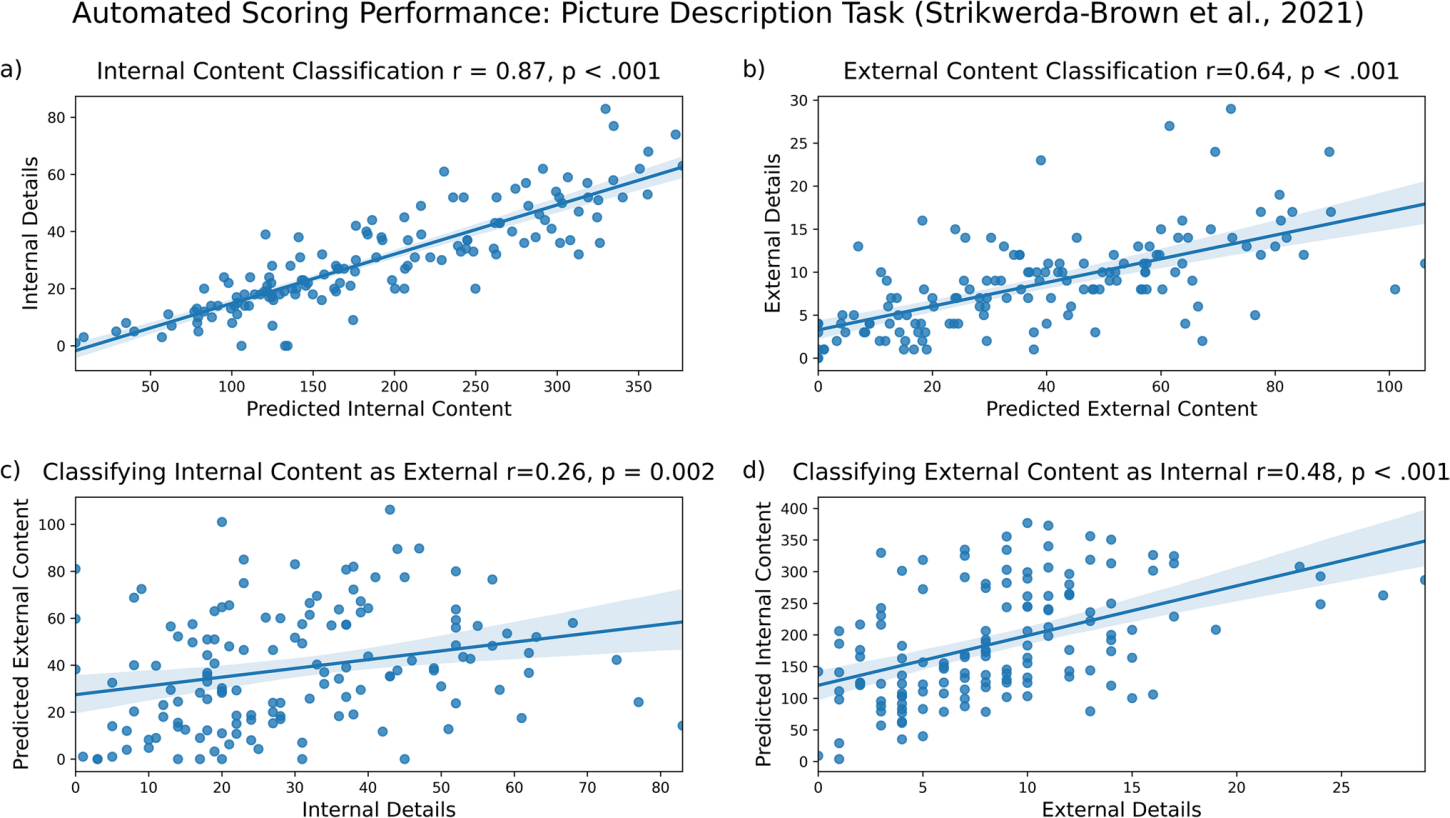
Model performance on picture description data from Strikwerda-Brown et al. (2021). Internal (**a**) and external (**b**) content are accurately identified, with significant misclassification of internal details as external (**c**) and external details as internal (**d**)

Our model correctly identified much of the internal content (Fig. 8a; *r* = .87, *p* < 0.001) and external content (Fig. 8b, *r* = .64, *p* < 0.001) in picture descriptions. However, internal content was often misclassified as external content (Fig. 8c, *r* = .26, *p* = 0.002) and external content was often misclassified as internal content (Fig. 8d, *r* = .48, *p* < 0.001). While we found greater correct classification of internal content than misclassification (*R*^2^ = 0.76 vs. *R*^2^ = 0.07), and we found greater correct classification of external content than misclassification (*R*^2^ = 0.40 vs. *R*^2^ = 0.23), misclassification is frequent. These results are summarized in the figure below.

#### Results: Thoughts task (Strikwerda-Brown et al., 2021)

**Fig. 9:**
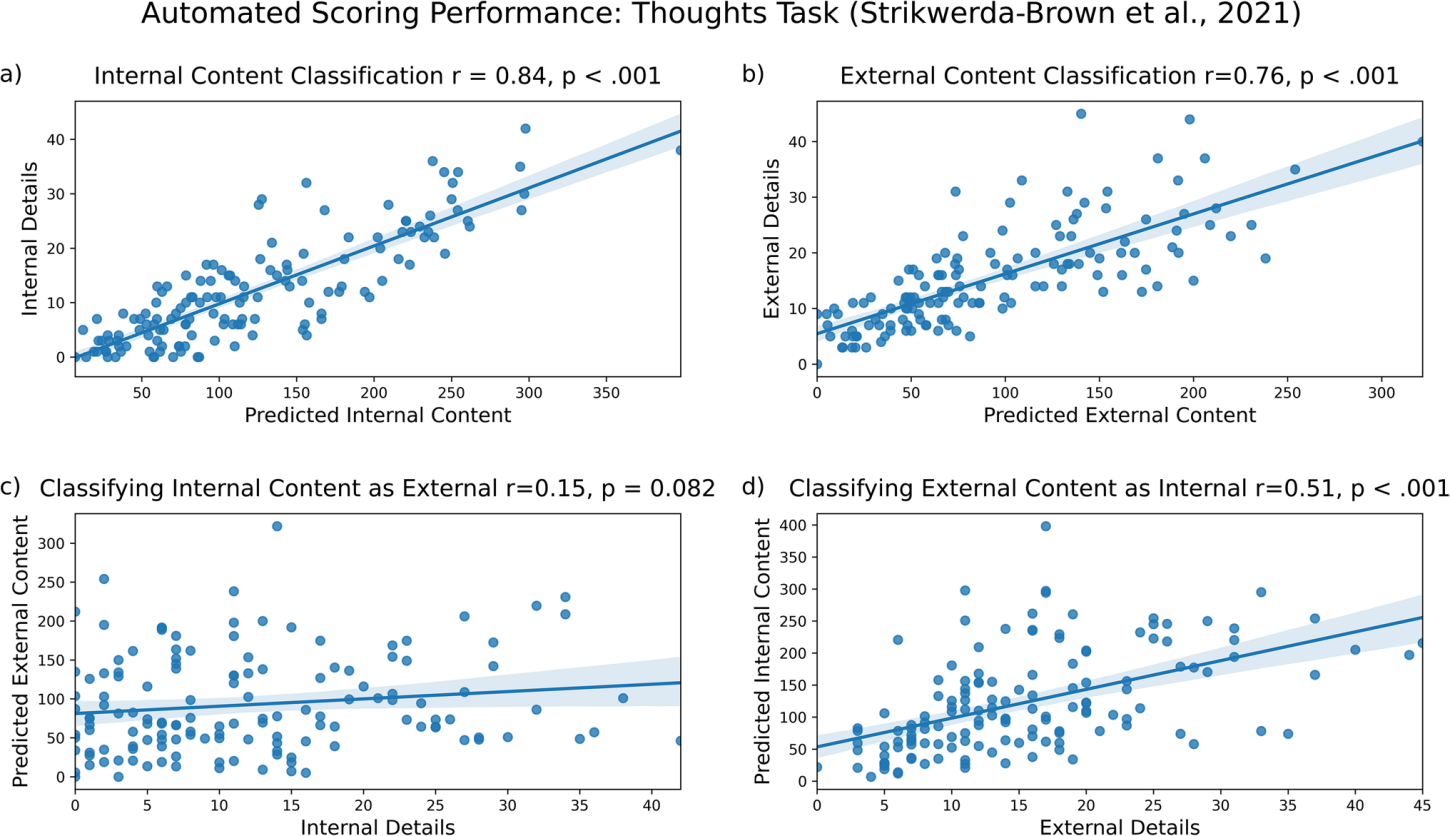
Model performance on thoughts task data from Strikwerda-Brown et al. (2021). Internal (**a**) and external (**b**) content are accurately identified, with much misclassification of internal details as external (**c**) and external details as internal (**d**)

When participants describe their unconstrained thoughts, our model correctly identified much of the internal content (Fig. 9a; *r* = .84, *p* <.001) and external content (Fig. 9b, *r* = .76, *p* <.001) in the resulting transcribed narratives. Significant misclassification was present. Internal content was not significantly misclassified as external content (Fig. 9c, *r* = .15, *p* < 0.082), but external content was often misclassified as internal content (Fig. 9d, *r* = .51, *p* < 0.001). To summarize, even though we found greater correct classification of internal content than misclassification (*R*^2^ = 0.71 vs. *R*^2^ = 0.02) and greater correct classification of external content than misclassification (*R*^2^ = 0.58 vs. *R*^2^ = 0.26), we observed significant misclassification. These results are summarized in the figure below.

## Discussion

We have described and tested a model-based approach that can automatically score memories, imagined events, and related narrative output for internal and external content. In general, we found that our model performs well across datasets with a variety of tasks in both older and younger populations. The amount of predicted internal content is highly correlated with actual internal detail counts in narratives. Likewise, the amount of predicted external content is highly correlated with actual external detail counts. Importantly, in most of the datasets content misclassification is relatively low: the number of internal details has little relationship to the amount of predicted external content. Likewise, there is no strong relationship between the number of external details and predicted internal content.

However, we found that model performance differed across datasets. Model performance was very good for future simulation narratives from older adults (Devitt & Schacter, 2018; Devitt & Schacter, 2020), creative writing narratives (van Genugten et al., 2021), and memory narratives from Strikwerda-Brown et al. (2021) and King et al. (2022). For these datasets, there was little misclassification of content. For musically cued memories (Sheldon et al., 2020), however, we found rates of misclassification that were higher than in the other datasets. Model performance substantially improved after manually adding punctuation. Together, these results suggest that performance of our model is good across datasets when punctuation is present in narratives.

We expected performance to be worse on the two tasks scored with different guidelines, because the model was not trained to mimic those guidelines. Indeed, performance was comparatively poor on the thoughts task, in which participants provided the thoughts that came to mind as they looked at a picture, and also on the related picture description task. These results suggest that our automated scoring procedure should not be used, or used with caution, on tasks that are not optimized for scoring with the interview (Levine et al., 2002) or the adapted interview (e.g., Addis et al., 2008).

### Optimal setting and potential limitations

Our model will likely perform best when used with data that are similar to our training data, i.e., when scoring internal and external data from future simulation and autobiographical memory tasks. We believe that the model can also be used for scoring other narrative data, as evidenced by strong performance on the creative writing dataset. However, we do not know how well this code will perform under new circumstances. For example, while the model seems to work well with data from both healthy young and older adults with relatively intact speech, its use with patient populations and populations with more rambling speech is untested. Researchers who want to use this automated scoring approach for new populations should manually score a subset of narratives to verify reliability. We are also unsure how well this model will perform for different dialects and for different language usage more generally. The datasets that we used for fine-tuning and evaluation were collected in the United States and Canada and thus were presumably from WEIRD (Henrich et al., 2010) populations. The data used for training distilBERT (the model we fine-tuned) comes from English Wikipedia text and the bookCorpus (a set of unpublished English books). Accordingly, narratives that use language that is significantly different from the English text found in our fine-tuning datasets or in the pretraining data may or may not be scored as accurately. Researchers who want to use this procedure in new populations can manually score a subset of narratives to confirm accuracy.

There are several situations in which we expect the model to score text differently from the standard interview procedure. First, we expect our code to improperly score narratives that do not have punctuation to mark sentence boundaries, as discussed earlier. Second, we expect narratives that contain several events to be scored differently from the standard interview procedure. With the typical interview scoring, researchers identify a central event, and mark all details in non-central events as external. The current code is not able to identify which details belong to central versus peripheral events. As a result, the model is likely to identify event-related details as internal, regardless of which event the details came from. Depending on the research question, this feature may or may not matter. Researchers interested in the total amount of episodic and non-episodic content in narratives can use the code as is, while researchers interested in only the central event may have to manually read the narratives and score a subset of them by hand.

### Recommendations for study design

We provide several design recommendations to maximize the power to detect an effect when using our automated scoring approach. First, we recommend using narratives with relatively clean text. Transcribed responses will sometimes contain meaningless text (e.g., ‘Hmmm…I don’t know, we went to… I guess, well…), which should be removed prior to automatic scoring. If this text is scored, it will add noise to our internal and external content measures. In addition, since our tool operates by first splitting text into sentences based on punctuation, researchers should include as much punctuation as is reasonable when transcribing. Clean text can also be obtained by asking participants to write out their responses, but we should note that there are limitations of this approach. Participants may be more concise and provide fewer external details in written responses when compared to verbal responses and may be limited by factors such as typing proficiency. Second, to achieve the same power as a manually scored study, studies using our procedure should recruit larger sample sizes. Third, prompting by the researcher (e.g., ‘Is there anything more you can tell me?’) should be removed from the transcript before it is automatically scored, otherwise these prompts will be automatically scored as internal or external content.

## Future directions

### Model modifications

In our work, we fine-tuned distilBERT for classification. Other neural network architectures that perform better on transfer learning tasks could be used in future work. Future work could also systematically search for different hyperparameters to improve classification accuracy. We used default hyperparameters for fine-tuning distilBERT. Last, we intend to test a different classification approach in future work. Our current model determines what percentage of content in each sentence is internal or external. In future work, we will fine-tune a model that classifies individual tokens, while taking into account context from surrounding text. By operating at the level of individual tokens (roughly equivalent to individual words) rather than at the sentence level, we expect greater accuracy. Importantly, this approach may eliminate the need to clean up transcripts from verbal responses, since uninformative speech tokens (e.g., ‘uhh’) could be classified as irrelevant based on annotation from existing datasets. Token classification would eliminate the need to split text into sentences as well, allowing run-on sentences to be transcribed without additional punctuation. Last, classifying text at the individual token level will facilitate subcategory scoring and enable time-series analyses (for applications, see e.g., Diamond & Levine, 2020).

### Scoring internal and external detail subcategories

The approach that we have presented here is useful for automatically calculating the amount of internal and external content in narratives. A second function of the interview is to sort internal and external content into further subcategories. Internal details can be further classified as perceptual, event, time, place, and thought/emotion related. External details can be classified as event (event details from non-central events), semantic (general knowledge or facts), repetition, and other content such as metacognitive statements and editorializing (for more detailed external subcategories, see Renoult et al., 2020; Strikwerda-Brown et al., 2019). The same approach that we used for classifying internal and external content could be extended for classifying detail subcategories. That is, researchers could train models to determine what percentage of content in each sentence belongs to each detail subcategory. In an alternative approach, we are currently fine-tuning a model to classify which subcategory each token comes from.

Standard interview administration also involves gathering subjective ratings for each memory from both participants and scorers, which evaluate the perceptual richness, time localization, place localization, and other qualitative aspects of each memory. Future work by other researchers could train a model on pairs of ratings and memories to automate this component of the interview as well.

## Conclusion and application

We believe that the tool presented here will enable researchers to conduct studies with considerably larger sample size than typically used, and thus perhaps capture smaller effect sizes, using the interview. We believe this tool will also facilitate Internet-based research with the interview. This type of research has often been impractical because of the scoring burden that comes from collecting more participants to offset data quality. Internet-based research would allow researchers to study more diverse populations and would allow memory researchers to take advantage of strategies used in other areas of psychology, such as rapidly piloting multiple experiments online. Importantly, the automated scoring procedure will enable research groups that have fewer resources for scoring narratives to also conduct large studies with the autobiographical interview.

To accompany this paper, we provide a Colab notebook that researchers can open in their web browser. Researchers can use this notebook to automatically score memories by providing a spreadsheet with narratives. The notebook is intended to be useable out-of-the-box without any additional coding required. This notebook and instructions for using it can be found at https://github.com/rubenvangenugten/. We also provide an example data spreadsheet there to guide users in formatting their data before running the code. The final model used in this notebook has been retrained on all adapted and standard interview-scored datasets.

### Supplementary Information

ESM 1 (DOCX 3.67 MB)
