# Supplementary material for: Automated scoring of the autobiographical interview with natural language processing
Source: Behav Res Methods. 2024 Jan 17;56(3):2243–59. doi: 10.3758/s13428-023-02145-x (PMC10990986; doi:10.3758/s13428-023-02145-x)
Supplement: Supplementary file 1 — (DOCX 3.67 MB) [file 13428_2023_2145_MOESM1_ESM.docx]

**Appendix**

**Example scoring**

During the autobiographical interview, a participant might provide a response such as the following:

We went to the beach because it was my birthday. I was sitting on the beach with a beer. We had driven up last night. We ran into the surf.

After applying the rules described in the scoring manual, we obtained the scored response below. We used forward slashes to separate details and we have surrounded the external details with brackets.

[We went to the beach because it was my birthday/]. I was sitting on the beach/ with a beer /. [We had driven up last night/]. We ran into the surf/.

We then counted the details to summarize the narrative: 2 external details and 3 internal details. If we were using our automated approach, we would summarize the narrative by counting the number of words in external segments (16 words in the brackets) and internal segments (14 words outside of the brackets).

**Separating text into details: an extended discussion**

For additional clarity, we provide an extended discussion on the relationship between internal details and internal word counts, the relationship between external details and external word counts, and in particular how these two relationships differ. Following this discussion, we discuss the robustness of these relationships for narratives with different proportions of internal and external content. Finally, we present an additional test of whether internal and external word counts serve as good proxies for detail counts.

***Internal and External Detail-Word Count Relationships***

The autobiographical interview provides different rules for identifying internal and external details, which lead to differences in the relationship between word counts and detail counts. External details often consist of general facts that are scored as one unit (e.g. ‘I’ve always liked going to the beach for my birthday’). As a result, external details frequently contain a relatively large number of words. Internal details are descriptors of event details, which can be much shorter (e.g. ‘brown’ in ‘a brown hat’ modifies a perceptual detail, and is therefore scored as its own internal detail). Therefore, external details generally contain more words than internal details.

To further illustrate this point, we show the relationship between internal details and internal word counts in an example dataset below (Strikwerda-Brown et al., 2021). The term “internal word count” refers to the number of words in all internal details of a narrative. To the right of this plot, we show the relationship between external details and external word counts. “external word count” refers to the number of words in all external details of a narrative.


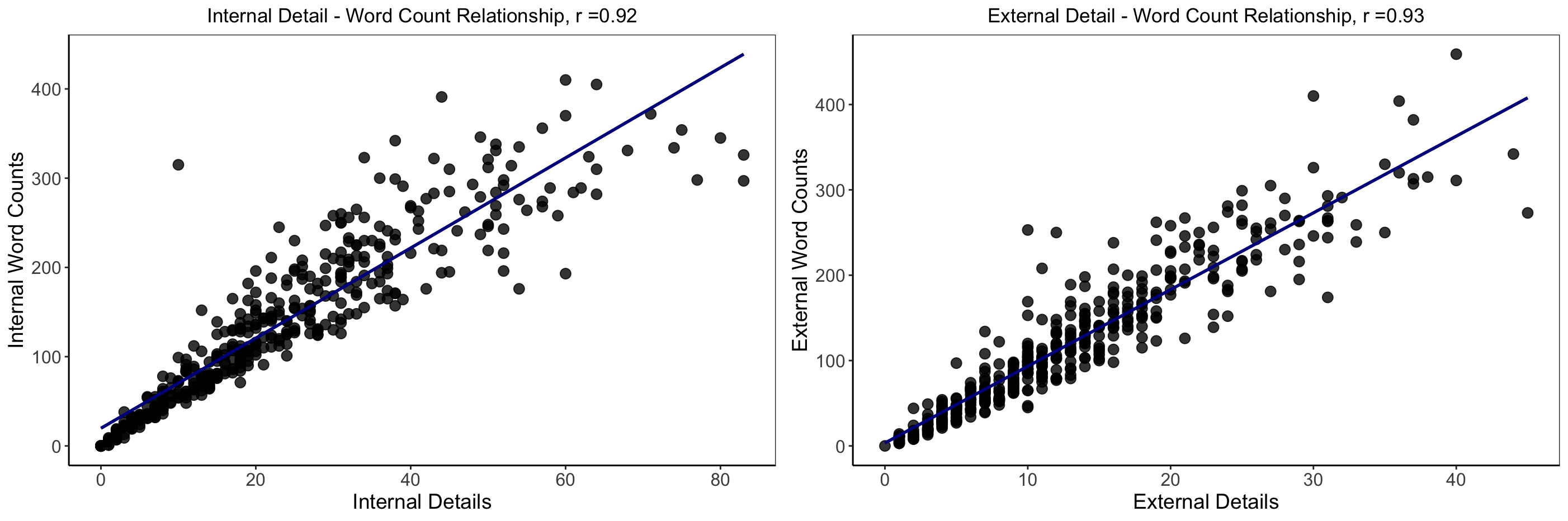


**Fig S1. Detail and word count relationships** in memory retrieval data from Strikwerda-Brown et al (2021). The relationship between internal details and internal word counts is displayed on the left, while the relationship between external details and external word counts is displayed on the right.

This dataset exhibits a strong correlation for both internal and external details with their respective word counts (r = .92, r = .93). However, the magnitude of the slope differs between the two plots (beta = 5.74 for internal content, beta = 8.14 for external content), indicating that each increase in external detail count is associated with a greater number of additional words when compared to each increase in internal detail.

For a thorough discussion of this topic, we should note that there is substantial variation between labs and raters in the relative coarseness of scoring internal and external content. We present an additional example dataset to demonstrate this point, using older adult data (Devitt & Schacter, 2018; 2020). In this dataset, the correlation between detail counts and associated word counts remains strong (r = .9 for internal content and r = .96 for external content). Yet, the number of words per detail do not differ substantially between internal and external content. As illustrated in Fig S2, forty internal details contain approximately 200 internal words. Likewise, 40 external details contain approximately 200 external words.

**
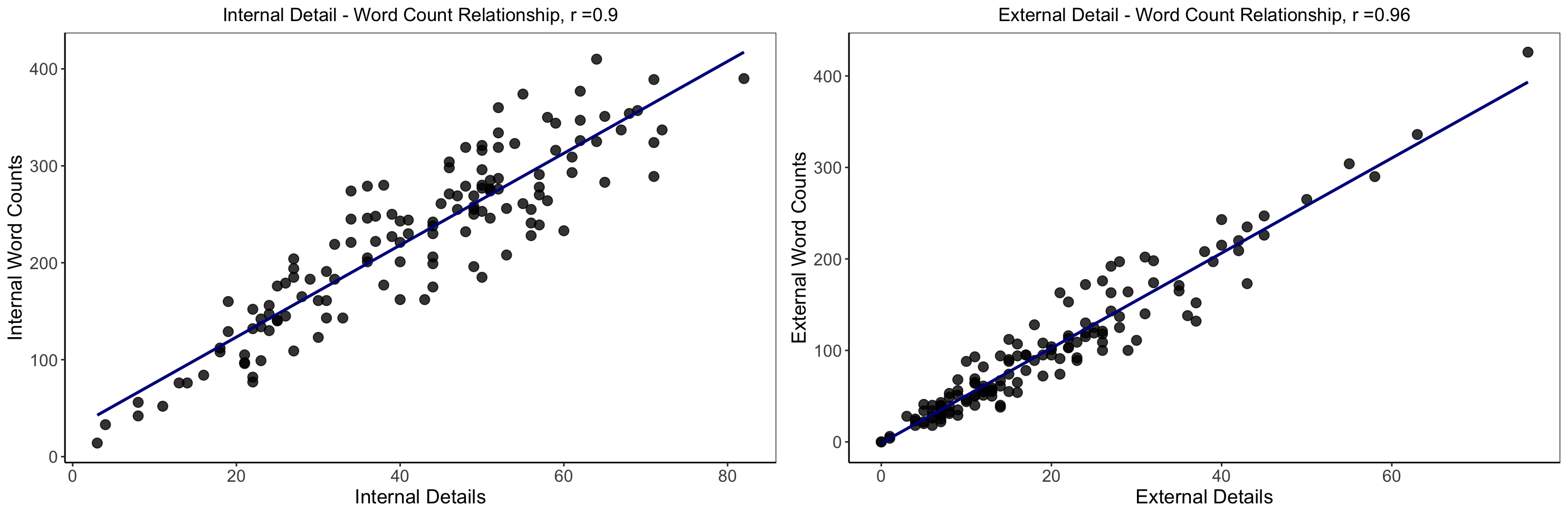
**

**Fig S2. Detail and word count relationships** in data from older adults in Devitt & Schacter (2018, 2020). The relationship between internal details and internal word counts is displayed on the left, while the relationship between external details and external word counts is displayed on the right.

Variation between labs in scoring leads to differences in detail segmentation (i.e. variation in the number of words per detail), yet the correlation between detail counts and associated word counts is strong across datasets. This provides an additional motivation for using internal and external word counts as proxies for detail counts, as they capture the relationships we are interested in, yet are robust to lab differences in how finely details are separated when they are scored.

***Robustness of Detail-Word Count Relationships***

To evaluate the robustness of word-detail count relationships, we tested whether these relationships remained strong across narratives with different proportions of internal and external content. To conduct this analysis, we first calculated the proportion of internal content in each narrative by dividing the number of internal details by the total number of details. For each dataset, we then sorted the narratives in ascending order based on the proportion of internal content they contained and divided them into quartiles. These four groups represented narratives with different levels of internal content proportions: narratives with the lowest 25% of internal proportion, 25th-50th percentile, 50th-75th percentile, and the top 25% of narratives based on proportion of internal content. For each quartile, we computed the correlation between internal details and internal word count, as well as the correlation between external details and external word count. Our analysis indicates that these correlations remain strong across groups of narratives with different proportions of internal content. This is most readily seen through the column averages in Table S1 and S2 below, which summarize the correlations as a function of internal proportion across datasets.

| Dataset | All | 0-25% | 25-50% | 50-75% | 75-100% |
| --- | --- | --- | --- | --- | --- |
| Sheldon et al. (2020) | .90 | 0.97 | 0.97 | 0.94 | 0.97 |
| Devitt & Schacter (2018, 2020) YA | .89 | 0.91 | 0.87 | 0.86 | 0.75 |
| Devitt & Schacter (2018, 2020) OA | .90 | 0.95 | 0.72 | 0.68 | 0.86 |
| Van Genugten et al. (2021) | .98 | 0.85 | 0.84 | 0.87 | 0.87 |
| Strikwerda-Brown et al. (2021) | .92 | 0.96 | 0.79 | 0.90 | 0.85 |
| King et al. (2021) | .96 | 0.94 | 0.96 | 0.93 | 0.96 |
| **Column Average** | **.93** | **0.93** | **0.86** | **0.86** | **0.87** |

**Table S1**. **Correlations between internal detail count and internal word count**, calculated for each quartile of narratives. Quartiles are based on the proportion of each narrative that consists of internal content. Correlations in the “All” data column are calculated from the full datasets.

| Dataset | All | 0-25% | 25-50% | 50-75% | 75-100% |
| --- | --- | --- | --- | --- | --- |
| Sheldon et al. (2020) | .88 | 0.97 | 0.95 | 0.95 | 0.97 |
| Devitt & Schacter (2018, 2020) YA | .96 | 0.93 | 0.78 | 0.70 | 0.86 |
| Devitt & Schacter (2018, 2020) OA | .96 | 0.87 | 0.82 | 0.77 | 0.88 |
| Van Genugten et al. (2021) | .98 | 0.70 | 0.79 | 0.81 | 0.91 |
| Strikwerda-Brown et al. (2021) | .93 | 0.88 | 0.88 | 0.88 | 0.93 |
| King et al. (2021) | .94 | 0.92 | 0.84 | 0.75 | 0.82 |
| **Column Average** | **.94** | **0.88** | **0.84** | **0.81** | **0.89** |

**Table S2**. **Correlations between external detail count and external word count**, calculated for each quartile of narratives. Quartiles are based on the proportion of each narrative that consists of internal content. Correlations in the “All” data column are calculated from the full datasets.

We do not observe systematic variation in the correlations as a function of the proportion of internal content in narratives. Because internal proportion and external proportion are directly related (e.g., if 20% of narrative details are internal, then 80% must be external), this also means that we observe no systematic variation in the correlations as a function of the proportion of external content in narratives.

***Further Validation of Word Counts as Detail Proxies***

To further examine the validity of using internal and external word counts as proxies for detail counts, we evaluated the effects of substituting detail counts with word counts. Specifically, we examined whether narratives maintained their rank order when detail counts were substituted with word counts. A preserved rank order would indicate that that the variables are interchangeable, and that internal and external word counts indeed function as good proxy variables for detail counts. To conduct these analyses for each dataset, we, we calculated narrative ranks based on internal details, calculated the narrative ranks based on internal words, and then calculated the correlation between the two rank vectors. High correlations in these analyses would suggest that narratives maintained their rank order when detail counts were replaced with their associated word counts. We repeated this analysis for external details. Results for two example datasets are presented in scatterplots below, followed by a table summarizing rank-order robustness in each of the datasets used in the manuscript.


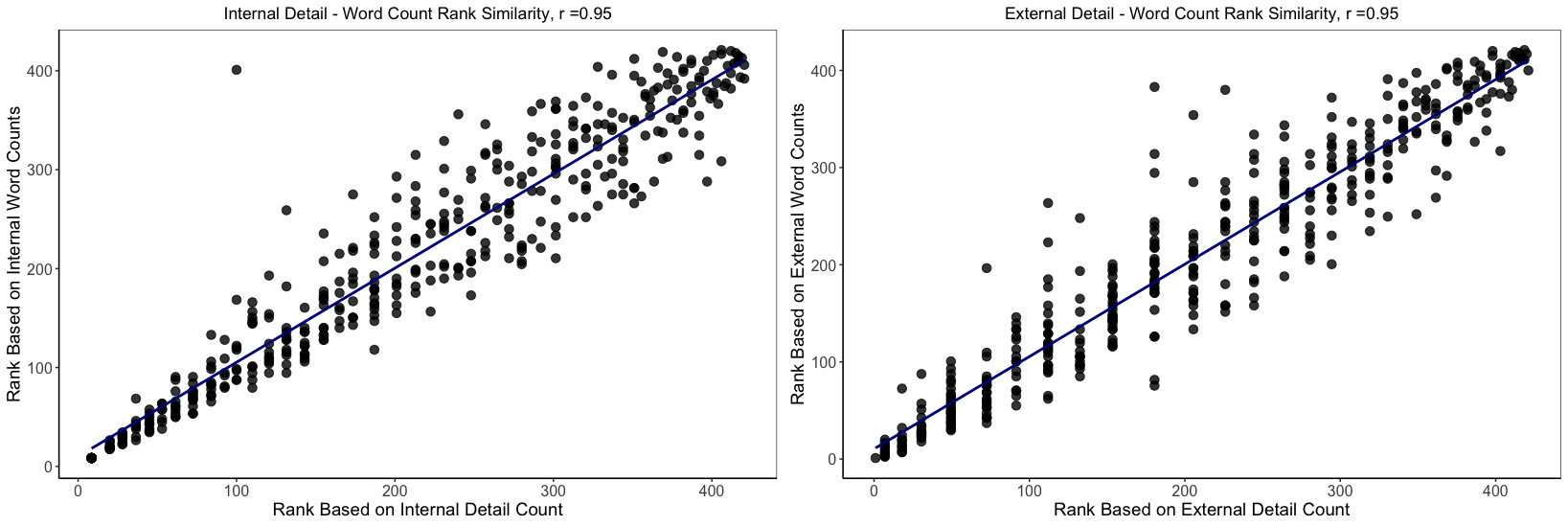


**Fig S3. Rank-order robustness of narratives** to replacing internal detail counts with internal word counts (left) in data from Strikwerda-Brown et al (2021). We also display the rank-order robustness of narratives to replacing external detail counts with external word counts (right). Strong correlations are present in both plots (r = .95), indicating rank-order robustness. Vertical stripes are seen in the plots because there are ties between detail counts when ranking data. For example, multiple narratives contain 5 external details, so these narratives all have the same rank on the x axis.

To further illustrate rank-order robustness of narratives to replacing detail counts with word counts, we display results in a second dataset below (older adult data from Devitt & Schacter, 2018; 2020).


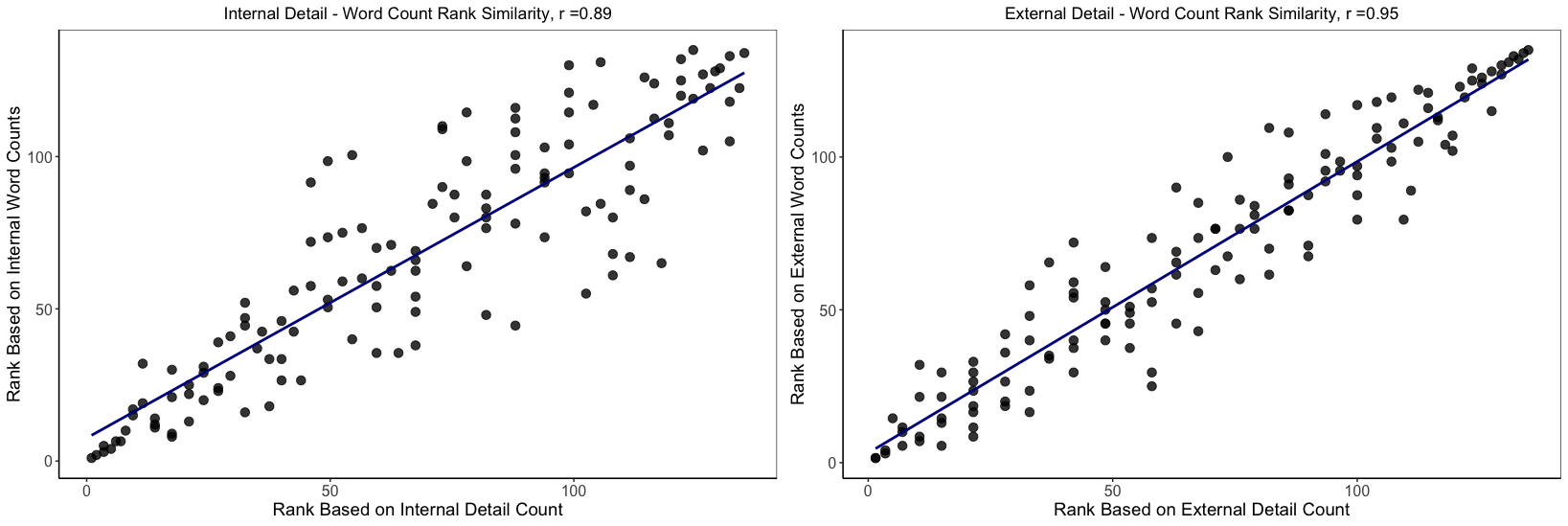


**Fig S4. Rank-order robustness of narratives** to replacing internal detail counts with internal word counts (left) in data from Devitt & Schacter (2018; 2020). We also display the rank-order robustness of narratives to replacing external detail counts with external word counts (right).

We summarize the relationship between rank order based on word counts and rank order based on detail counts in each of the datasets in the table below. We find that rank-order is largely preserved, as indicated by consistently high correlations.

| Dataset | Rank-ordered  internal content  correlation | Rank-ordered  external content  correlation |
| --- | --- | --- |
| Sheldon et al. (2020) | 0.90 | 0.92 |
| Devitt & Schacter (2018, 2020) YA | 0.88 | 0.96 |
| Devitt & Schacter (2018, 2020) OA | 0.89 | 0.95 |
| Van Genugten et al. (2021) | 0.98 | 0.98 |
| Strikwerda-Brown et al. (2021) | 0.95 | 0.95 |
| King et al. (2022) | 0.96 | 0.94 |

**Table S3. Rank-order robustness of narratives** to replacing detail counts with word counts**.** “Rank-ordered internal content correlation” refers to the correlation between the narrative rank based on internal detail count and the narrative rank based on internal word count. “Rank-ordered external content correlation” refers to the correlation between the narrative rank based on external detail count and the narrative rank based on external word count.

These analyses suggest that narrative ordering is robust to replacing detail counts with word counts, which further suggests that word counts can serve as a strong proxy for detail counts.

***Outlier analysis***

In the main manuscript, we noted that data from King et al. (2022) contained several very long narratives. It is important to emphasize that these outliers reflect talkative participants rather than faulty data. In this section, we examined whether our results are robust to the removal of these outliers by assessing model performance after outlier exclusion. To remove outliers, we calculated the mean number of internal details in narratives, then removed narratives with three standard deviations more internal details. We repeated this procedure for external details. In total, we removed 4.5% of narratives (﻿24 out of 524 narratives). Scoring performance after the removal of outliers is summarized in the figure below. For completeness, we repeated the outlier analyses for all datasets and present those results in table S4.


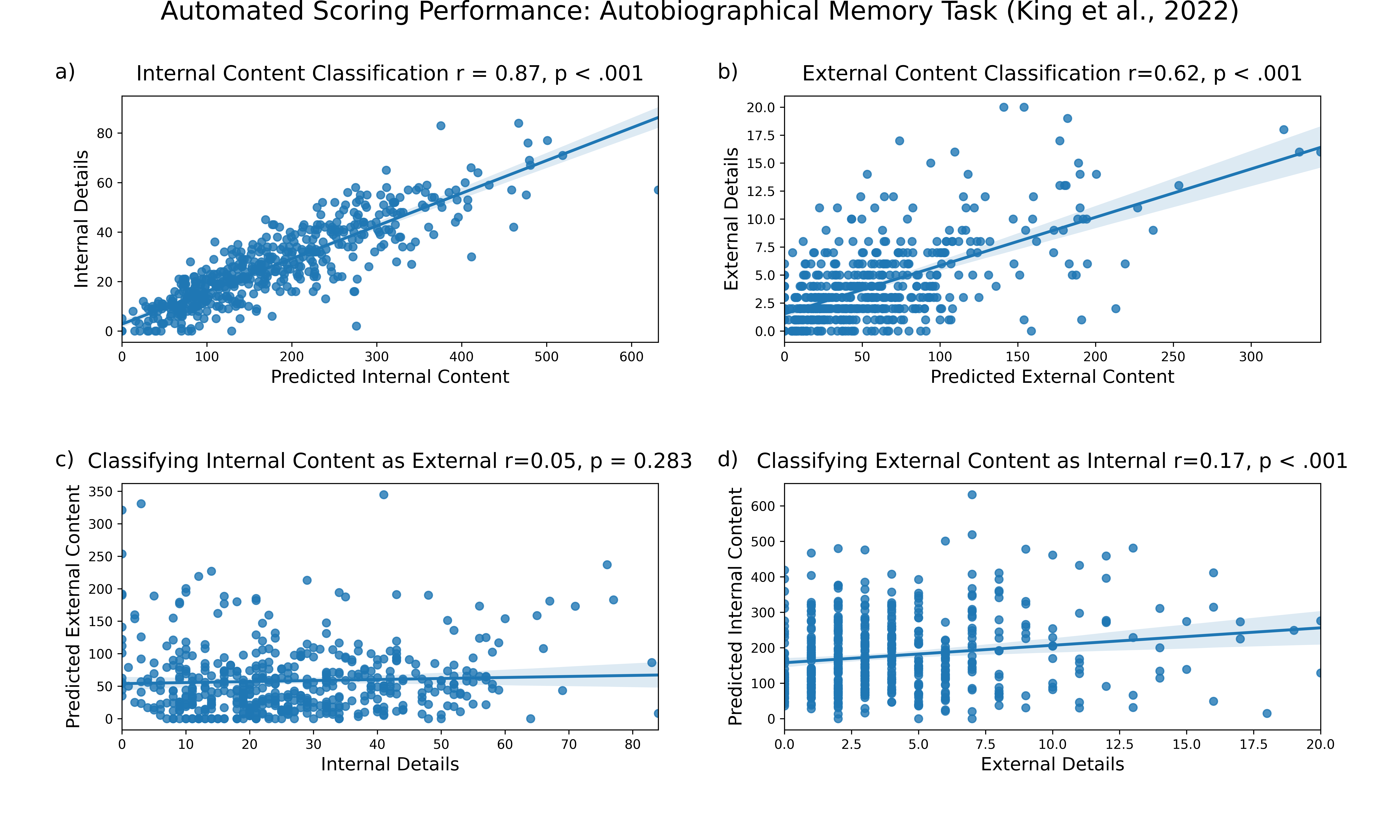


**Fig S5. Model performance on autobiographical memory data from King et al. (2022) after removal of outliers**. Internal (panel A) and external (panel B) content are accurately identified, with minimal misclassification of internal details as external (panel C) and external details as internal (panel D).

| Dataset | Change Cor. A | Change Cor. B | Change  Cor. C | Change Cor. C | Percent  Data Removed |
| --- | --- | --- | --- | --- | --- |
| Sheldon et al. (2020) | 0.00 | -0.03 | -0.10 | -0.03 | 3.2 |
| Devitt & Schacter (2018, 2020) YA | 0.02 | 0.03 | 0.02 | 0.00 | 1.4 |
| Devitt & Schacter (2018, 2020) OA | 0.00 | 0.00 | 0.02 | 0.02 | 0.7 |
| Van Genugten et al. (2021) | -0.04 | -0.01 | 0.00 | 0.01 | 2.6 |
| Strikwerda-Brown et al. (2021) | 0.01 | 0.01 | 0.01 | 0.00 | 0.7 |
| King et al. (2021) | -0.02 | -0.11 | 0.10 | 0.00 | 4.5 |

**Table S4. Change in automated scoring performance after outlier removal.** Model performance is summarized by four correlations throughout this paper: (a) accurate internal identification: the correlation between predicted internal content and actual internal detail counts, (b) accurate external identification: the correlation between predicted external content and external detail counts, (c) classifying internal content as external: the correlation between internal details and predicted external content, and (d) classifying external content as internal: the correlation between external details and predicted internal content. In this table, we report how these four correlations change after outlier removal. The last column describes the percent of narratives that are removed from each dataset using the procedures described earlier.

Our analyses show that automated scoring performance changes little after outliers are removed.

**Data preprocessing: additional discussion**

When applying this tool to new datasets, we encourage users to carefully transcribe narratives with extensive punctuation. Considering the substantial amount of data included in our analyses (totaling 596,678 words across datasets), we chose to introduce a preprocessing step to produce datasets resembling those that have been carefully transcribed, rather than manually check punctuation in all of our text. Specifically, we excluded longer sentences, defined as those containing more than eight details. For data transparency, we discuss the amount of text affected by preprocessing. For most datasets, minimal text removal occurred. In written responses from King et al. (2021), 0.6% of sentences were removed by this procedure. We removed 2.9% of younger adult data and 7.4% of older adult data from transcribed responses of Devitt & Schacter (2018; 2020). For transcribed responses in Sheldon et al. (2020), 2.4% of data was excluded. Two datasets had a more considerable proportion of text removed. As highlighted in the results section, a significant portion of the data in Strikwerda-Brown et al. (2021) was transcribed with limited punctuation. This is not a problem for manual scoring but makes automated scoring difficult. With this preprocessing procedure, 20.5% of data was removed to create an artificial dataset mimicking one with more punctuation. In the results section, we showed that manually adding punctuation to a random subset of this text (without preprocessing) improved performance. Finally, a significant amount of data was removed during preprocessing of written responses from van Genugten et al. (2022). This dataset was scored according to an adapted Autobiographical Interview manual that divided text into a greater number of details than the original scoring manual would. As a result, the detail count per sentence is inflated relative to other datasets. When making our artificial dataset, 29.4% of sentences were removed during preprocessing.

**Open Practices Statement**

Code for automated scoring can be found at https://github.com/rubenvangenugten/autobiographical_interview_scoring. This repository also contains the code used to fine-tune our language model, as well as code for producing the plots in this paper. The final model is hosted at <https://huggingface.co/vangenugtenr/autobiographical_interview_scoring>. Data used in this paper cannot be publicly shared due to regulations from various IRB protocols. Analyses presented in this paper were not preregistered.

**References**

Addis, D. R., Wong, A. T., & Schacter, D. L. (2008). Age-related changes in the episodic simulation of future events. *Psychological Science, 19*, 33–41. https://doi.org/10.1111/j.1467-9280.2008.02043.x

Azunre, P. (2021). *Transfer learning for natural language processing*. Simon and Schuster.

Devitt, A. L., & Schacter, D. L. (2018). An optimistic outlook creates a rosy past: the impact of episodic simulation on subsequent memory. *Psychological Science, 29*, 936–946. https://doi.org/10.1177/0956797617753936

Devitt, A. L., & Schacter, D. L. (2020). Looking on the bright side: aging and the impact of emotional future simulation on subsequent memory. *The Journals of Gerontology: Series B, 75*, 1831–1840. https://doi.org/10.1093/geronb/gbz041

Devlin, J., Chang, M.-W., Lee, K., & Toutanova, K. (2019). BERT: Pre-training of deep bidirectional transformers for language understanding. *ArXiv*. http://arxiv.org/abs/1810.04805

Diamond, N. B., & Levine, B. (2020). Linking detail to temporal structure in naturalistic-event recall. *Psychological Science*, *31*, 1557-1572.

Gaesser, B., Sacchetti, D. C., Addis, D. R., & Schacter, D. L. (2011). Characterizing age-related changes in remembering the past and imagining the future. *Psychology and Aging, 26*, 80–84. https://doi.org/10.1037/a0021054

Henrich, J., Heine, S. J., & Norenzayan, A. (2010). The weirdest people in the world?. *Behavioral and Brain Sciences, 33*, 61-83.

Irish, M., Hornberger, M., Lah, S., Miller, L., Pengas, G., Nestor, P. J., Hodges, J. R., & Piguet, O. (2011). Profiles of recent autobiographical memory retrieval in semantic dementia, behavioural-variant frontotemporal dementia, and Alzheimer’s disease. *Neuropsychologia, 49*, 2694–2702. https://doi.org/10.1016/j.neuropsychologia.2011.05.017

Jing, H. G., Madore, K. P., & Schacter, D. L. (2016). Worrying about the future: An episodic specificity induction impacts problem solving, reappraisal, and well-being. *Journal of Experimental Psychology: General*, *145*, 402.

King, C.I., Romero, A.S.L., Schacter, D.L., & St. Jacques, P.L. (2022). The influence of shifting perspective on episodic and semantic details during autobiographical memory recall. *Memory*, *30(8)*, 942-954. https://doi.org/10.1080/09658211.2022.2061003

Levine, B. (2021) Memory. The Levine Lab. Retrieved November 9, 2021 from <https://levinelab.weebly.com/memory.html>

Levine, B., Svoboda, E., Hay, J. F., Winocur, G., & Moscovitch, M. (2002). Aging and autobiographical memory: Dissociating episodic from semantic retrieval. *Psychology and Aging, 17*, 677–689. https://doi.org/10.1037/0882-7974.17.4.677

Liu, Y., Ott, M., Goyal, N., Du, J., Joshi, M., Chen, D., Levy, O., Lewis, M., Zettlemoyer, L., & Stoyanov, V. (2019). RoBERTa: A robustly optimized bert pretraining approach. *ArXiv*. http://arxiv.org/abs/1907.11692

Madore, K. P., Gaesser, B., & Schacter, D. L. (2014). Constructive episodic simulation: Dissociable effects of a specificity induction on remembering, imagining, and describing in young and older adults. *Journal of Experimental Psychology: Learning, Memory, and Cognition, 40*, 609.

Madore, K. P., & Schacter, D. L. (2014). An episodic specificity induction enhances means-end problem solving in young and older adults. *Psychology and Aging, 29*, 913.

Peters, J., Wiehler, A., & Bromberg, U. (2017). Quantitative text feature analysis of autobiographical interview data: Prediction of episodic details, semantic details and temporal discounting. *Scientific Reports, 7*, 14989. https://doi.org/10.1038/s41598-017-14433-6

Race, E., Keane, M. M., & Verfaellie, M. (2011). Medial temporal lobe damage causes deficits in episodic memory and episodic future thinking not attributable to deficits in narrative construction. *Journal of Neuroscience, 31*, 10262–10269. https://doi.org/10.1523/JNEUROSCI.1145-11.2011

Renoult, L., Armson, M. J., Diamond, N. B., Fan, C. L., Jeyakumar, N., Levesque, L., ... & Levine, B. (2020). Classification of general and personal semantic details in the Autobiographical Interview. *Neuropsychologia*, *144*, 107501.

Sadvilkar, N., & Neumann, M. (2020). PySBD: Pragmatic sentence boundary disambiguation. *ArXiv*. http://arxiv.org/abs/2010.09657

Sanh, V., Debut, L., Chaumond, J., & Wolf, T. (2019). DistilBERT, a distilled version of BERT: Smaller, faster, cheaper and lighter. *ArXiv*. ArXiv:1910.01108.

Schacter, D. L., & Addis, D. R. (2007). The cognitive neuroscience of constructive memory: remembering the past and imagining the future. *Philosophical Transactions of the Royal Society of London B: Biological Sciences, 362*, 773-786.

Schacter, D. L., & Madore, K. P. (2016). Remembering the past and imagining the future: Identifying and enhancing the contribution of episodic memory. Memory Studies, *9*, 245–255.

Sheldon, S., Williams, K., Harrington, S., & Otto, A. R. (2020). Emotional cue effects on accessing and elaborating upon autobiographical memories. *Cognition*, *198*, 104217. https://doi.org/10.1016/j.cognition.2020.104217

Söderlund, H., Moscovitch, M., Kumar, N., Daskalakis, Z., Flint, A., Herrmann, N., & Levine, B. (2014). Autobiographical episodic memory in major depressive disorder. *Journal of Abnormal Psychology*, *123*, 51–60. https://doi.org/10.1037/a0035610

Strikwerda-Brown, C., Mothakunnel, A., Hodges, J. R., Piguet, O., & Irish, M. (2019). External details revisited – A new taxonomy for coding ‘non-episodic’ content during autobiographical memory retrieval. *Journal of Neuropsychology*, *13*, 371–397. https://doi.org/10.1111/jnp.12160

Strikwerda-Brown, C., Williams, K., Lévesque, M., Brambati, S., & Sheldon, S. (2021). What are your thoughts? Exploring age-related changes in episodic and semantic autobiographical content on an open-ended retrieval task. Memory, 29(10), 1375–1383. https://doi.org/10.1080/09658211.2021.1987476

Takano, K., Gutenbrunner, C., Martens, K., Salmon, K., & Raes, F. (2018). Computerized scoring algorithms for the Autobiographical Memory Test. *Psychological Assessment*, *30*(2), 259–273. https://doi.org/10.1037/pas0000472

Takano, K., Hallford, D. J., Vanderveren, E., Austin, D. W., & Raes, F. (2019). The computerized scoring algorithm for the autobiographical memory test: Updates and extensions for analyzing memories of English-speaking adults. *Memory*, *27*, 306–313. https://doi.org/10.1080/09658211.2018.1507042

Takano, K., Ueno, M., Moriya, J., Mori, M., Nishiguchi, Y., & Raes, F. (2017). Unraveling the linguistic nature of specific autobiographical memories using a computerized classification algorithm. *Behavior Research Methods*, *49*, 835–852. https://doi.org/10.3758/s13428-016-0753-x

van Genugten, R. D., Beaty, R. E., Madore, K. P., & Schacter, D. L. (2021). Does episodic retrieval contribute to creative writing? an exploratory study. *Creativity Research Journal*, 1–14.

Wardell, V., Esposito, C. L., Madan, C. R., & Palombo, D. J. (2021a). Semi-automated transcription and scoring of autobiographical memory narratives. *Behavior Research Methods*, *53*, 507–517. https://doi.org/10.3758/s13428-020-01437-w

Wardell, V., Madan, C. R., Jameson, T. J., Cocquyt, C. M., Checknita, K., Liu, H., & Palombo, D. J. (2021b). How emotion influences the details recalled in autobiographical memory. *Applied Cognitive Psychology*, *35*, 1454–1465. https://doi.org/10.1002/acp.3877

Wickner, C., Englert, C., Addis, D.R. (2015). Developing a tool for autobiographical interview scoring. Kiwicam Conference, Wellington, New Zealand. https://github.com/scientific- tool-set/scitos

Williams, J. M., & Broadbent, K. (1986). Autobiographical memory in suicide attempters. *Journal of Abnormal Psychology*, *95*, 144–149. https://doi.org/10.1037/0021-843X.95.2.144

Wolf, T., Debut, L., Sanh, V., Chaumond, J., Delangue, C., Moi, A., Cistac, P., Rault, T., Louf, R., Funtowicz, M., Davison, J., Shleifer, S., von Platen, P., Ma, C., Jernite, Y., Plu, J., Xu, C., Le Scao, T., Gugger, S., … Rush, A. (2020). Transformers: State-of-the-Art natural language processing. *Proceedings of the 2020 Conference on Empirical Methods in Natural Language Processing: System Demonstrations*, 38–45. https://doi.org/10.18653/v1/2020.emnlp-demos.6

Yin, W., Hay, J., & Roth, D. (2019). Benchmarking zero-shot text classification: Datasets, evaluation and entailment approach. *ArXiv*. http://arxiv.org/abs/1909.00161
